# Effect of mechanical stirring on sonoluminescence and sonochemiluminescence

**DOI:** 10.1016/j.ultsonch.2024.107145

**Published:** 2024-11-06

**Authors:** Atiyeh Aghelmaleki, Hossein Afarideh, Carlos Cairós, Rachel Pflieger, Robert Mettin

**Affiliations:** a Department of Energy Engineering and Physics, Amirkabir University of Technology, 424 Hafez Avenue, 15875-4413 Tehran, Iran; b Third Institute of Physics, Georg-August-University Göttingen, Friedrich-Hund-Platz 1, 37077 Göttingen, Germany; c Institute for Separation Chemistry of Marcoule (ICSM), Univ Montpellier, CEA, CNRS, ENSCM, Marcoule, BP 17171, 30207 Bagnols sur Cèze Cedex, France

**Keywords:** Sonoreactor, Stirring, Sonoluminescence, Ethylene glycol, Phosphoric acid, Gas diffusion

## Abstract

Light emissions from cavitating liquids serve as a diagnostic tool for chemical activity, bubble collapse conditions, or excited species. Here we demonstrate the influence of mechanical stirring on sonoluminescence (SL) and sonochemiluminescence (SCL) emissions emerging in the presence of dissolved sodium salts and luminol in different sonicated liquids. In the systems investigated, driven in the 20–40 kHz range, stirring can change the spatial distribution of blue/white broadband SL emissions and of the orange sodium D-line emission, as well as their relative intensities. In many cases, an amplification of sodium emission is observed under stirring, but striking exceptions appear as well. SCL emission from luminol is mainly quenched by the mechanical agitation. The liquids under study comprise water, ethylene glycol, and phosphoric acid, all with dissolved argon or krypton, and partly xenon. From the stirring-induced changes in sonoluminescence and from high-speed video recordings, we try to draw conclusions on the actual effects of the induced rapid bulk liquid flow on the cavitating systems. Main effects seem to be enhanced gas diffusion and bubble separation or de-clustering, possibly by a separative impact of the stirring flow on populations of smaller and larger bubbles, respectively. As a consequence, large bubbles with extremely bright sonoluminescence flashes appear in phosphoric acid under stirring. Overall, a certain sensitivity of stirring effects on further system parameters has to be stated.

## Highlights

• Mechanical stirring can influence spectra and spatial distributions of sonoluminescence and sonochemiluminescence.
• Effects are observed for different acoustic fields, liquids, and dissolved gases.
• The physical mechanisms comprise enhanced gas diffusion, bubble de-clustering, and redistribution of nuclei.
• Stirring in phosphoric acid can lead to individual extremely bright sonoluminescing bubbles.

## Graphical abstract

**Figure:**
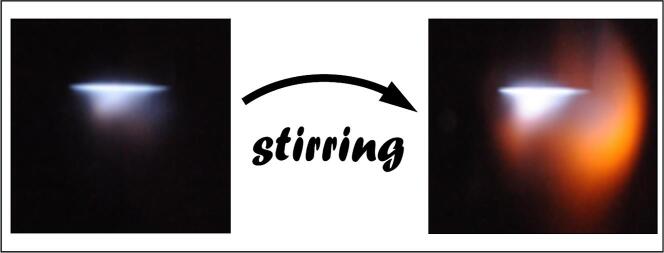

## 1 Introduction

In sonochemistry, intense ultrasound and acoustic cavitation are applied to trigger, enhance or steer chemical reactions and chemical processes in liquid media. The variety of applications is immense, and the particular mechanism of impact of the cavitation bubbles can be quite diverse. As a popular tool for diagnosis and design of reactors, light emissions in form of sonoluminescence and sonochemiluminescence are frequently used, since they can relatively easily map, qualify, and partly quantify the chemical activity of cavitation bubbles.

Sonoluminescence (SL) is known as the emission of light from collapsing cavitation bubbles in a liquid [1], [2], [3]. The light emerges from the hot, partly ionized compressed interior of the imploded bubbles and is plasma emission, i.e. a combination of black body radiation, recombination radiation, bremsstrahlung, and emissions from excited species [3], [4], [5], [6]. Sonochemiluminescence (SCL), in contrast to SL, arises in the liquid phase from secondary reactions of dissolved species. Typically, hydroxyl radicals (OH°) react with dissolved luminol molecules resulting in the emission of blue light [7], [8], [9]. It is worth noting that parameter regimes, bubble populations or bubble dynamics of active SL and SCL emissions can significantly differ, see for instance [10], [11], [12].

Mechanical stirring as a way to agitate liquid is a very common and possibly the oldest method to enhance heat and mass transport in liquid phase reactions [13]. Therefore, it is straightforward to consider it as an addition to sonochemical reactors for enhancement of reaction rates or yields. However, the impact of stronger bulk liquid flow on acoustic cavitation is not simple, as several aspects of such rather parameter sensitive systems are affected [14]. For instance, nuclei and bubbles can be redistributed in space, gas diffusion, gas dissolution, or degassing from the liquid can be stimulated, shear forces in the liquid might split bubbles and multiply them, quasi-static pressure drop could be induced, and more.

In the past, the influence of stirring on cavitating systems has been investigated mainly through the changes in sonochemical reaction yields, but also via luminescence emissions. With respect to chemical yields, different effects have been observed in several studies. Most results showed an effective increase in radical production or reaction efficiency when ultrasound was combined with stirring or with an externally driven flow [15], [16], [17], [18], [19], [20], [21], [22], while other results documented a decrease in sonochemical yields or an irrelevant effect [23], [24], [20], [21]. For instance, Yasuda *et al.* [17] reported that stirring with a turbine increased sonochemical activity at 486 kHz, while the effect of a propeller was negative in KI dosimetry, positive in the degradation of tetraphenylporphine tetrasulfonic acid. These at first sight contradictory results actually reflect the complex interaction between an agitated bulk flow and many other factors influencing sonochemical reactions, like acoustic frequency and power, temperature, dissolved gas, type of transducer and geometrical design of the sonoreactor, and so on. The involved combination of all these factors must necessarily have an effect on cavitation bubble dynamics and/or characteristics of bubble collapses, correlated with intra bubble conditions. Bussemaker et al. in their work [20], [21] systematically studied the effect of overhead stirring and external flow in cavitation fields at high and low frequencies under air atmosphere. They found that stirring enhances OH radical production at low frequencies, but has the contrary effect at high frequencies. As an illustrative result coming out of this work, the maximum yield in radical production was obtained at 40 kHz combined with overhead stirring, which is contrary to the frequent experience that higher frequencies (300–900 kHz) are more efficient in radical production (see, e.g., [19]). This shows the versatility of ultrasonic cavitation in function of the different variables that might affect cavitation bubble dynamics, and demonstrates that many parameters might be invoked for optimization of a system. Bussemaker et al. speculate on a decrease in bubble coalescence as the main reason behind an increase in radical yield at low frequencies, but no clear experimental corroboration was reported. Further suggested factors influenced by stirring or flow include an acoustic travelling wave vs. standing wave field, and a disturbed sphericity of bubble collapse, see the discussion in Bussemaker et al. [20], [21]. Furthermore, stirring effects can be sensitive to the dissolved gas. Pflieger *et al.* [19] have found an increase in H_2_O_2_ accumulation under stirring during Ar-O_2_ sparging at higher frequencies (200, 362 and 600 kHz), but rather a decrease under pure Ar sparging (not published).

An effect of mechanical agitation or imposed liquid flow on SL and SCL has also been reported. Hatanaka *et al.* [25], [26] found different effects of the liquid flow on SL in air-saturated distilled water at low and high power in standing-wave conditions at 23 and 99 kHz. At low power, SL intensity decreased when stirring or liquid flow were applied. On the contrary, at high power, corresponding to the power when SL intensity started to decrease in the absence of stirring (correlating to the appearance of bubble clusters moving away from the pressure antinodes), stirring led to a strong increase in SL intensity. The latter increase was attributed to the observed disappearance of the bubble clusters, destroyed by the liquid flow, and the appearance of streamers. As for luminol SCL, it could generally be amplified [25] by stirring up to some power at frequencies from 23 to 131 kHz. For 99 kHz, SCL was shown to be less sensitive to stirring at low powers, but strongly increased with stirring speed at high powers. Kojima et al. [18] found an extension of the luminol SCL emission zone when stirring was applied in a 490 kHz reactor, but the overall SCL intensity was reported to decrease at the same time. The sonochemical yields, however, were increased by stirring.

Regarding spectral characteristics of SL emissions, changes have been observed by Pflieger *et al.* [27] that were attributed to the perturbing flow of rising sparging bubbles. Differences in the population of electronic and rovibronic states of OH radicals occurred depending on whether measured SL bubbles were collapsing freely or close to the rising bubbles, apparently being mechanically perturbed by the gas flow. The spectral differences indicated higher excitation, i.e. more extreme intra-bubble conditions, in the unperturbed case.

As the principal reasons for the effects of agitation on chemistry and SL in a cavitation field, phenomena involving bubble cloud characteristics and bubble interactions, such as bubble coalescence or bubble clustering, have been conjectured [25], [26], [20]. Nevertheless, direct support of such effects, for instance by high-speed imaging, are sparse [25], [26], and several other mechanisms might be involved, as mentioned. Furthermore, all previous studies employed overall measures of SL intensity or SCL spatial mapping, and to the best of our knowledge, no studies apart from [27] have been reported so far on the influence of external flow or stirring on the different emission lines or other spectral characteristics of the UV–Vis spectrum of SL or SCL. This will be addressed in the present work, in particular since spectral information can give additional clues on the influence and type of perturbation on the bubble dynamics, caused by flow agitation.

In the spectrum of “native” multibubble-SL (i.e. emerging directly from the strong compression of the bubble interior), one can classify broadband (continuum) and line (atomic and molecular) emissions. Their relative intensities depend on several parameters, and in particular on the collapse symmetry. Xu *et al.* and others described essentially two different active cavitation bubble populations [28], [29], [30], [31], [32]: (1) Rather stationary bubbles of smaller size and with highly symmetric collapse, and (2) often larger bubbles that collapse non-spherically and/or oscillate with strong surface deformations, caused by instabilities or rapid motion. In sonotrode setups, population (1) is rather found close to the transducer tip, while population (2) is often fast moving in a streaming liquid flow outside of the dense clouds of the population (1). It is conjectured that SL continuum emissions, as well as emissions from species formed inside the bubble core from volatile components, like OH, emerge predominantly from the first group of bubbles. In contrast, SL emissions from non-volatile components are supposed to be facilitated via injection of liquid micro- or nano-droplets into the gas phase of the collapsing bubbles [28], [33], and this requires significant deformation from sphericity, as found for the second bubble group. For instance, the emissions from excited alkali metal atoms, as well as from the PO radical that is a decomposition product from nonvolatile H_3_PO_4_ molecules, are expected to origin rather from the second group of cavitating bubbles. Mechanical agitation like stirring usually generates shear flows and turbulence that potentially influence the bubble dynamics and perturb the spherical symmetry. Thus, it would not be surprising if stirring shifts the bubble populations towards the second group of bubbles.

In this work, we investigated the effects of mechanical stirring on the spatial distribution of SL and SCL under low frequency sonication, i.e. in the range of 20 kHz to 40 kHz. We worked in three different liquids (de-ionized (DI) water, ethylene glycol (EG), and phosphoric acid (PA)) with dissolved sodium salts and with different dissolved noble gases (Ar, Kr, and for direct sonoluminescing bubble observations with Xe). Further, also luminol solutions were probed. Overall UV–Vis emission spectra were recorded in part of the experiments to corroborate visual impressions, detect line emissions and measure line-to-continuum ratios.

A description of the experimental details is given in Section 2. The results are presented in Section 3 for three distinct liquids: water (Section 3.1), ethylene glycol (Section 3.2), and phosphoric acid (Section 3.3), where also observations from high-speed recordings are included. Subsequently, a discussion of the results is provided in Section 4, followed by a conclusion in Section 5.

## 2 Experimental setups and methods

**Fig. 1:**
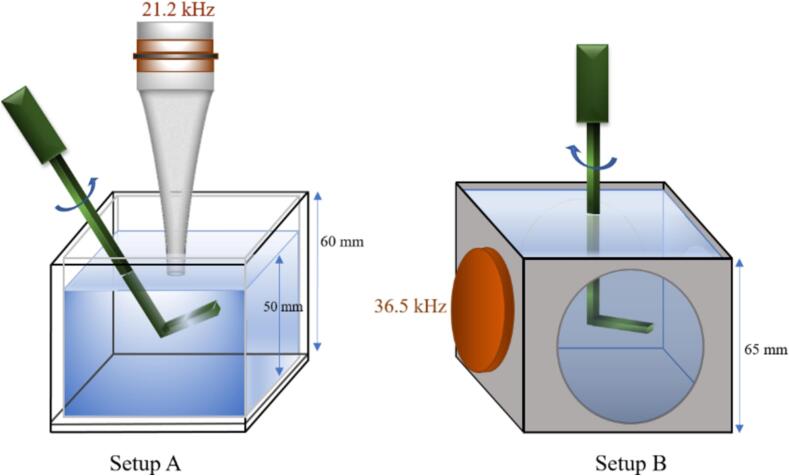
Schematic sketch of the two setups. Left: setup A with a cubic PMMA cuvette of 6 cm edge length, horn transducer from top driven with 21.2 kHz, stirrer obliquely from top. Right: setup B with a cubic stainless-steel cuvette of 6.5 cm edge length, piezo plate transducer from the side driven at 36.5 kHz, stirrer vertically from top. Gas sparging took place via the bottom plate.

Two experimental setups were used to investigate the influence of stirring on sonoluminescence and sonochemiluminescence in the different liquids, see Fig. 1. In some cases, spectroscopic data in the UV–Vis region were also collected.

Setup A is an in-house-built cubic PMMA cuvette of 6 cm side length, sonicated from the open top via an in-house-built sonotrode (exponentially shaped titanium horn with circular tip of 1  cm diameter, 0.79  cm^2^ area), operating at 21.2 kHz, and submerged to 1  cm liquid depth. The water level was maintained at 5  cm in all experiments conducted with setup A. Setup B is an in-house-built 6.5  cm side length cubic stainless steel reactor driven from one side by an attached Langevin transducer at 36.5 kHz (5  cm diameter, 19.63  cm^2^ area), with three (5 cm diameter) quartz windows on the other lateral sides of the cuvette. All acoustic transducers were driven by HF power amplifiers (E&I 1040 L, USA) via an impedance matching coil (in-house-built) at the indicated fixed driving frequency, which was delivered by a frequency generator (Tektronix AFG 3021, USA). The indicated sonication power is given as the real electric input power as read from the amplifier, and values for the reported results ranged from 10  W to 20  W. Nominal delivered power densities ranged accordingly from 12.73 to 25.46  W/cm^2^ at 21.2  kHz and from 0.51 to 1.02 W/cm^2^ kHz at 36.5  kHz.

As a stirring element for setups A and B, a mechanical stirrer (Heidolph IKA-Euro ST 20 D, Germany) with a rotation frequency range of n=0-2000 rotations per minute (rpm) was hosted through the open top of the reactors. Its rotation center was held at a constant depth of 2 cm (A) and 3.5 cm (B) below the free surface of the liquids (both reactors were open to the atmosphere). In the following, we will refer to the rotation frequency or rotation rate n, given in rpm, also as “rotation speed”, which is customary in the literature. The impeller blade was of simple L-form (one-sided blade of length R≈2 cm and thickness b≈1 mm), and the actual velocity of the outer impeller blade tip in the laboratory frame results from the expression $v_{h}=2\pi Rn$. The indicated rpm ranges result in tip velocities $v_{h}$ from zero to about 4 m/s, which reaches the fastest measured bubble translation velocities from high-speed recordings in setup A (not shown here). The Reynolds numbers of the impeller head is typically defined [13] as Re=nd2ρ/μ=2vhRρ/(πμ) with the impeller head diameter d=2R (ρ and μ being the liquid density and dynamic viscosity, respectively). In our experiments we reach up to about Re≈50000 for water and Re≈3400⋯4250 for PA and EG. This shows that all cases are expected to be turbulent at highest impeller speeds, but that the non-aqueous systems remain close to the transition from laminar circulation due to the higher viscosities. From high-speed movies (not shown), we infer that the impeller is producing mainly a radial flow pattern (sideways), which is, however, superimposed by the downwards directed jet flow generated by the sonotrode.

Three different liquids with different viscosities and vapor pressures were used in the experiments. Several potentially relevant physico-chemical and acoustic properties are given in Table1.

**Table 1:** Liquid properties of DI water, pure and 95 vol% ethylene glycol (EG) in water and phosphoric acid (PA), as far as available. Data sources indicated; the values for 95 vol% EG have been obtained by interpolation / extrapolation of data from the references.

***liquids***	***dynamic viscosity*** μ (cPoise)	***density*** ρ (g/cm^3^)	***sound speed*** c (m/s)	***surface tension*** γ (N/m)	***vapor pressure*** p_v_ (Pa)	***solubility of gases in liquid*** Henry's-law constant K_H_ (GPa)		
						***Ar***	***Kr***	***Xe***
***water***	1.00 (20 °C) [34]	0.998 (20 °C) [34]	1493 (20 °C) [34]	0.072 (20 °C) [34]	2310 (20 °C) [35]	4.0 (25 °C) [36]	2.203 (25 °C) [37]	1.316 (25 °C) [37]
***ethylene glycol*** ***(pure)***	18.376 (20 °C) [35]	1.114 (20 °C) [35]	1660 (25 °C) [34]	0.0477 (25 °C) [34]	11 (20 °C) [35]	1.134 (25 °C) [38]	0.896 (20 °C) [39]	0.151 (25 °C) [38] 0.098 (20 °C) [39]
***ethylene glycol*** ***(95 vol%)***	13.1 (26.7 °C) [40]	1.13 (26.7°C) [40]	≈ 1660 (25 °C) [41]	0.0473 (30 °C) [42]	338 (20 °C) [Raoult’s law]	−	−	−
***phosphoric acid*** ***(85 % aq. sol.)***	43.5 (25 °C) [43]	1.686 (25 °C) [43]	1646 [44]	0.0746 (20 °C) [45]	299 (20 °C) [43]	−	−	−

Different concentrations of NaCl (99.5 %, Sigma-Aldrich, USA) and Na_3_PO_4_ (99.9 %, Carl Roth GmbH, Germany) were used to prepare alkali metal salt solutions in deionized water (DI), ethylene glycol, and phosphoric acid. The used solutions are aqueous solutions of 0.1 mM or 3 M of NaCl, 0.3 M NaCl solutions in 95 vol% ethylene glycol (prepared with DI water from pure EG, 99.9 % Sigma-Aldrich, USA), and 0.1  M or 0.5  M sodium phosphate (Na_3_PO_4_) in 85 wt% phosphoric acid (99.9 % pure, Carl Roth GmbH, Germany). The sonochemiluminescence measurements for the aqueous NaCl solutions and ethylene glycol solutions were carried out with 0.1 mM luminol (3-aminophthalhydrazide 98 %, Fluka-USA) solution, using NaOH solution to change the pH of the liquids to 11.5. To work with luminol in EG in a basic environment, the pH of EG/luminol solution was adjusted by adding 32 % NaOH aqueous solution until a pH meter (paper strip) showed the color of 11–11.5.

For setup A, all solutions were vacuum degassed for 1 h and then sparged (prior to ultrasonic irradiation) for 45 min with a noble gas (argon, krypton, or xenon, 99.999 %, Sigma-Aldrich, USA). Gas solubilities in the different liquids are given in Table1, if available.

In setup B the liquids were sparged with krypton or xenon at a constant flow of 60 ml/min also during the experiment. The gas entered through a fixed hole in the bottom of the cuvette. In some runs, the liquids were cooled before the experiment, and their initial temperature at the beginning of the ultrasound treatment is reported. The temperature rise during ultrasound exposure reached about 0.5 °C per minute at the highest powers (20 W). The on-times were kept to a minimum (mainly the respective exposure times of camera or spectrometer after a few seconds transients) to avoid a stronger heating effect.

The spatial distribution of SL/SCL emission was captured with a DSLR camera (D700, Nikon, Japan) fitted with a 50  mm lens (AF Nikkor 50 1:1.4D, Nikon, Japan). The exposure times were 30  s or 5  min for the aqueous NaCl and EG solutions, and 1 or 5  s for the PA solutions.

Spectroscopic measurements were performed in the range of 250–700 nm with an Acton Research SP-300i imaging spectrometer coupled to a charge-coupled-device detector (PIXIS 100B, Princeton Instrument). The spectrometer was equipped with two gratings: 600 gr/mm blz. 300  nm and 300 gr/mm blz. 500  nm, with nominal resolutions of Δλ= 0.2 and 0.4  nm, respectively. The wavelength calibration was performed with a Hg(Ar) pen ray light source (LSP 035, LOT, Germany). In setup A, the spectrometer slit was placed directly next to the cuvette in a height slightly below the sonotrode tip, with the optical axis running horizontally about 0.5  cm below the tip. In setup B, the slit was put centrally directly next to the observing window. From NA specifications we estimate a spectrometer entrance cone of about 15° full angle. In setup A this translates into an observed region roughly covering the range from the sonotrode tip until about 1  cm downwards. In setup B, the region of interest expands to a central region of the cuvette of about 1  cm as well. All spectra were acquired at a slit width of 0.25 mm and corrected against the sensitivities of the gratings and CCD camera as provided by the manufacturers. The exposure time for recording spectra in the 3 M NaCl solutions in setup A was 5 min, and in phosphoric acid solutions in setup B it was 180 s. The uncertainties of the SL intensities for repeated experiments amounted to about 20 %, and for intensity ratios to about 35 %. We did not include error bars in the figures for better readability.

## 3 Results

Here we present results of the stirring effects on SL/SCL emissions, sorted by the different liquids. Further parameters that have been varied comprise the setups A and B (different geometries and frequencies), the applied power, type of dissolved gas, initial liquid temperature, the presence of alkali salts, and the presence of luminol. We note that not all possible combinations of parameters have been probed.

### 3.1 Deionized water

**Fig. 2:**
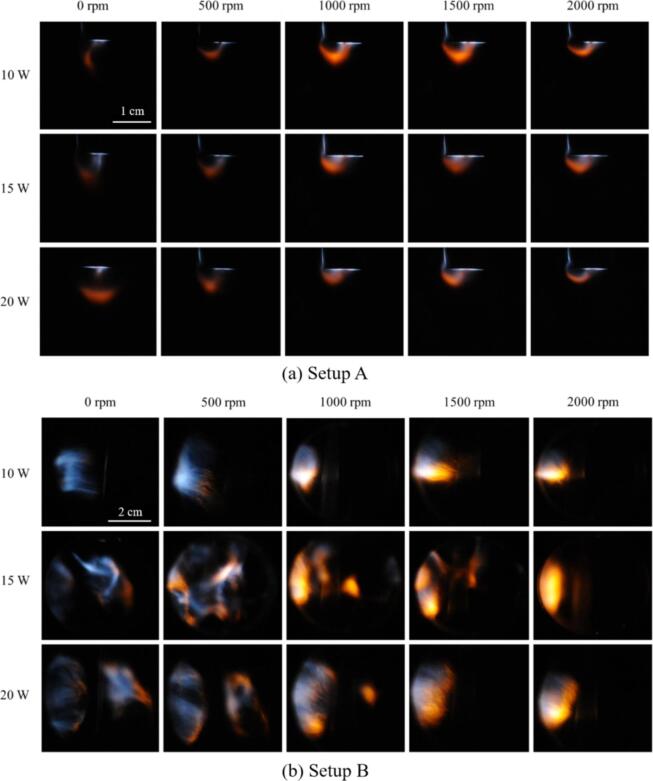
The spatial distribution of sonoluminescence in 3 M NaCl aqueous solution purged with Kr, at different acoustic powers and stirring velocities. (a) Setup A, 21.2 kHz, sonotrode tip visible on top; liquid temperature 13 °C, exposure time 5  min, frame width 3 cm. (b) Setup B, 36.5 kHz, transducer plate to the left; liquid temperature 20 °C, exposure time 30 s, frame width 5.4 cm.

**Fig. 3:**
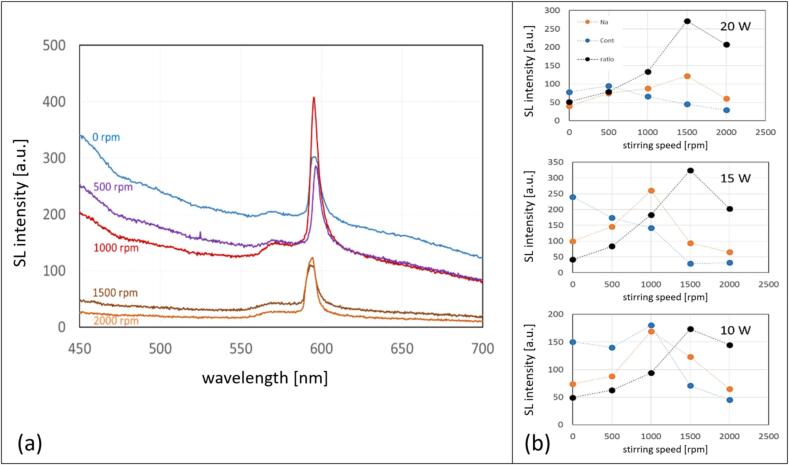
(a) The emission spectra in the visible region of the 3 M NaCl aqueous solution with Kr in setup B at 36.5 kHz and 15 W at different stirring velocities. (b) The changes in the sodium emission (589 nm – orange), the continuum emission (average over 450–480 nm − blue), and the ratio of sodium/continuum*100 (black), with respect to the stirring velocities (0–2000  rpm), and for three different acoustic powers of 10, 15 and 20 W, respectively.

Fig. 2 shows the SL emission of the 3 M NaCl aqueous solution saturated with Kr gas in setups A and B. Each column shows pictures at fixed stirring rates, obtained at the indicated electrical powers of 10, 15 and 20 W (rows). The power of 10 W lies slightly above the cavitation threshold (visible and/or audible cavitation events) for both systems, and 20 W is close to the maximum SL emission, which has been determined from previous trials (not shown here). In both setups, essentially two main colors of SL emission can be identified by eye: white-to-blue (“continuum”) and orange (“sodium”). These emission zones appear more or less spatially separated in both setups, although their borders touch or overlap, and the contribution of each color component is difficult to judge without spatially resolved spectral information. Further, the spectral composition could be varying in different depths, of course. From a rudimentary spatial color spectrum analysis, we conclude that orange emission can appear together with a significant share of blue, but also without (see Supplementary Material). Similar color separation or composition of two types of SL has been reported before, for instance in aqueous solutions by Refs. [46], [47], [48], [49], [50], and for acids by Refs. [51], [28], [32]. As already mentioned, it has been suggested that different bubble populations with different collapse dynamics are responsible for the effect [52], [53], [46], [47], and direct imaging of bubble dynamics support this interpretation [32], [54]. In brief, white-blue light emerges from bubbles of population (1) that collapse rather spherically, heat up strongly due to their symmetry, and do not significantly mix the liquid phase into the hot interior. Orange emission stems from excited sodium (Na*) and indicates a certain amount of liquid phase sprayed into the collapsing bubble (since the sodium ions are non-volatile and would otherwise be absent from the vapor phase). The entry of non-volatile compounds is usually attributed to non-spherical bubble dynamics, as shown by bubbles from the population (2). An asymmetry and non-spherical collapse can be caused by different factors like close bubble neighbors or objects, surface instabilities, or bubble motion [53], [55], [32], [54]. Non-preserved symmetry and liquid droplets inside the orange collapsing bubbles would suggest a weaker gas heating than in the white-blue bubbles, although further parameters like compression ratio should be taken into account for a conclusive statement. Here we report that mechanical stirring can influence the colored sonoluminescence phenomenon. Fig. 2 shows that not only the overall SL intensity can be changed [25], [26], but also the extension and location of the emission zones. In particular, an emitting zone can change its color, as can be seen, for instance, in Fig. 2b for the region next to the left side (in front of the transducer) that changes from blue to orange. Further analysis by emission spectra is given below in Fig. 3, Fig. 5.

In the absence of stirring, SL in setup A appears in a compact, round shaped emission region directly at and below the sonotrode tip (slightly off-centered at lower power), see Fig. 2a left column. One can distinguish mainly three different structures inside the emission region: a whitish flat horizontal layer directly at the tip, a blue-white jet structure downwards-sideways from the tip center, and an orange half-sphere or spherical cap structure farther from the tip. An additional blue region partly occurs at the lower end of the sonotrode as a vertical stripe. Increasing power leads to an intensification of the light and a certain extension of the SL region and its substructures, but the emissions nevertheless stay close to the sonotrode. This is partly different from the emission patterns reported for sulphuric acid by Eddingsaas et al. ([56], without sodium) and Xu et al. ([28], with sodium) that convert to a cone at the highest acoustic intensities of the horn (30 and 28  W/cm^2^), which occurs as well in water [57]. Probably we could not reach this regime for power limitations (< 25.5  W/ cm^2^). In setup B, left column of Fig. 2b, whitish-blue emission occurs in front of the transducer (left) as well. Moreover, orange areas appear on the far side (right), but only at elevated power. Under stronger driving, the emission region expands throughout the sonicated cell, but exhibits dark gaps, indicating a standing wave pattern (pressure nodes). Blue light is seen at higher power also far from the transducer, apparently around an acoustic pressure antinode. The colored SL patterns in Fig. 1a resemble observations reported before at a sonotrode for sulphuric acid [28], [58], [32], and the images in Fig. 2b compare to those from standing waves for aqueous systems [46], [48], [49], [50] and acids [30], [58]. While SL spectra of NaCl aqueous solutions at a sonotrode have been reported already by Lepoint-Mullie et al. [59], a direct color imaging of the cavitation zone has only been reported by Choi [31], where a fully orange conical zone appears. Thus our observations may be the first images of color separation of Na* emissions and blue continuum in an aqueous system employing a sonotrode setup. The fact that the shape of the orange emission region is quite similar to the acid case should indicate a similar distribution of bubble populations and collapse dynamics, in spite of the different liquids and therefore distinct viscosities and vapor pressures. This might help for better and unified interpretations of the underlying mechanisms.

#### 3.1.1 Stirring effects

When analyzing the impact of stirring, it seems obvious that some changes occur in both setups (columns 2 to 5 in Fig. 2a and b). Mechanical agitation has some influence not only on the spatial distribution of the light emission but also on the colors. Comparing emissions in the agitated setups A and B in detail, there appear certain similarities, as well as differences. In both cases, increased stirring speeds confine the SL activity to a region closer to the transducer: more to the sonotrode tip in A, and more to the left side wall in B (where the transducer plate is located). This retraction of emission zones ultimately leads to an almost extinction of the light from standing wave structures far from the transducer at high stirring rates in setup B. Furthermore, in both setups, a transition from blue emission to orange color appears with increased stirring velocities (apart from the zone directly at the sonotrode tip in setup A, where also an additional blue region partly occurs as a vertical stripe at the lower horn shaft). The shift to orange means that at strong agitation, the Na* emission seems to predominate over the continuum (white-blue) emission. We note that this trend resembles the increased Na* emission relative to the continuum observed with increasing Ar gas flow rate in (non-stirred) aqueous salt solutions [60].

Note that in setup B, increasing the power under constant stirring rate seems to extend the emission zones in most cases. For setup A, a notable SL amplifying effect of increasing power without stirring exists, but it is somehow reversed when stirring is applied: raising power at 1000  rpm rather reduces the visual intensity.

Differences between the setups appear in the overall extensions of SL emission regions, the amount of overlap or apparent interpenetration of the colors, and a stronger amplification of the orange regions by stirring in setup B. Part of these observations are probably related to the different geometries and the corresponding acoustic wave fields. A rough characterization of the acoustic source can be given by the parameter ka with k=2π/λ being the wavenumber, λ the acoustic wavelength, and a the transducer radius [61]. A sonotrode typically has low ka (ka≈0.44 in setup A) and resembles a point source, thus creates a high intensity spot right at the tip and a decaying field with high traveling wave and low standing wave contribution further away. The vertical piezo-ceramic plate in setup B has ka≈3.8 and is supposed to develop a stronger directivity, resulting in a field with dominant horizontal standing wave share and less traveling wave part. This can lead to distinct antinodes of high pressure amplitude and accordingly to SL activity far from the emitter, resulting in more extended and potentially more complex shaped SL regions. Additionally, setup B was continuously sparged with Kr gas, while setup A was only sparged before the run. This could contribute to the more intense colors in B. Assuming that a high pressure amplitude prevails near the transducers (and near the standing wave antinodes), and that it decays farther away, both setups agree with the observation that white-blue color arises from the high pressure zones, while the sodium signal is generated in the transition zones to lower pressure (i.e. at higher pressure gradients). This is in coincidence with the observations in other liquids, e.g. [30], [32]. In the present experiments, under applied stirring, the sodium emission regions can shift toward the higher pressure regions, and the blue emission zones are diminishing accordingly. At a medium power of 15 Watts in setup B, the isolated blue color tends to disappear completely, and at the maximum stirring speed of 2000 rpm, only a bright orange SL zone remains near the transducer (although we cannot exclude that blue bubbles exist even more left, closer to the transducer but outside the cuvette window region, or are covered by bright orange emission). Thus, the emission color has clearly changed just by the mechanical agitation. For a more detailed analysis of orange and blue regions in terms of RGB channel splitting, we refer to the Supplementary Material.

The UV–Vis SL emission spectra in the case of setup B confirm the visual observations. Fig. 3a shows the spectral evolution of the SL emission at 15 W for increasing stirring velocity (matching with the middle row in Fig. 2b). A continuum background, rising to the blue, is attributed to the white-blue emission, and a prominent peak around 589 nm represents the Na* emission (D-line doublet). The relatively broad peak or shoulder to the left at about 570 nm is usually attributed to the emission of the Na·Kr exciplex (the Na·Ar exciplex being nominally at 554 nm [59]). Otherwise, the spectra are essentially featureless.

Increasing the stirring speed leads to a reduction of the continuum part of the spectra, whereas the Na* peak remains and even grows to reach the highest value for 1000 rpm. A more detailed analysis of the relationship between the continuum and line emission is depicted in Fig. 3b for each acoustic power. As a measure of the blueish continuum, the average emission intensity in the range of 450 nm to 480 nm is taken (after background subtraction; blue points). For quantification of the line emission, the Na* peak maximum relative to the interpolated continuum is evaluated (orange points). The ratio between line and continuum “Na/cont” is calculated, multiplied by a factor of 100, and presented as black points. It is apparent that Na/cont grows monotonously with stirring up to a speed of 1500 rpm and then reduces again. Interestingly, the maximum value of the ratio of Na/cont appears uniquely at 1500 rpm, although the individual maximum values for the Na* and continuum emissions are different for all investigated acoustic powers.

#### 3.1.2 Noble gas effects

**Fig. 4:**
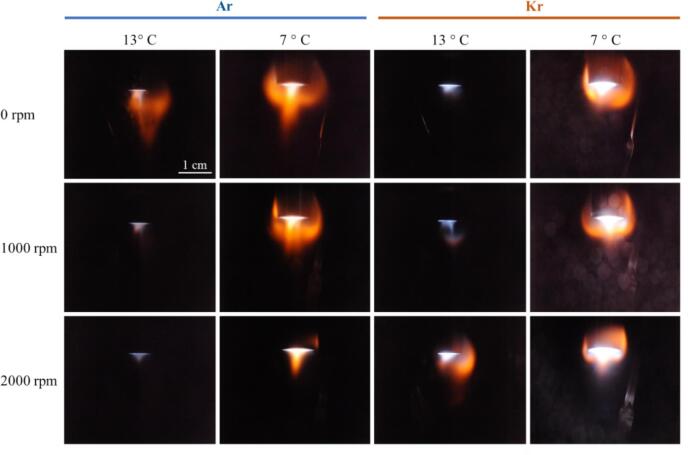
Spatial distribution of sonoluminescence in 3 M NaCl aqueous solutions purged with Ar and Kr, at high acoustic power (20 W) and varying stirring velocities in setup A (21.2 kHz, exposure time 5  min, temperatures and scale indicated). The linear features to the bottom right of the sonotrode tip are reflections from the metallic stirrer.

Further experiments regarding the effect of different noble gases, temperature, and the presence of luminol were done in setup A, always driven at 20  W. Fig. 4 compares SL emission at 0, 1000, and 2000 rpm stirring rate (rows) for four different 3 M NaCl aqueous solutions, being sparged with Ar and Kr, and at 13 °C and 7 °C. The runs for Kr at 13 °C from Fig. 2a (central row) have been repeated on another day and with longer exposure time (5  min), and the pictures differ slightly from those in Fig. 4. As before, the spatial SL distribution consists of a white-blue emission originating from a conical region right under the sonotrode tip, along with an orange emission emanating from a region farther around the sonotrode shaft. However, for Ar and for Kr at decreased temperature, Na* emission appears already prominently without stirring. Further, when stirring is applied, the effect on the color distribution of SL seems opposite for the two noble gases: Surprisingly, for increased agitation the orange regions diminish in presence of Ar, while for Kr they broaden (at 13 °C) or at least remain prominent (at 7 °C).

As well, an intensification of SL for reduced liquid temperature is observed, and this is in accord with previous reports for many gas–liquid combinations [3]. Here we see the temperature effect for “colored” SL in water, and it affects both, blue and orange regions. As an explanation, the reduced vapor pressure in the colder liquid is usually employed, leading to less trapped water molecules [62] and thus less cushioning of the bubble collapse, which results in higher collapse peak temperatures. The vapor pressure effect should be rather independent from the non-condensable gas in the bubble [63], which is coherent with the observations here. Stirring, on the other hand, seems to be selectively reducing Na* emission for Ar, enhancing Na* emission for Kr, and not affecting strongly the continuum emissions in both cases. The continuum background appears brighter for Kr than for Ar, which again agrees with previous observations, and which should mainly be due to the lower heat conduction of the heavier noble gas, lower ionization potential which increases the intensity of plasma emissions, and its higher solubility [64], [65], [3].

**Fig. 5:**
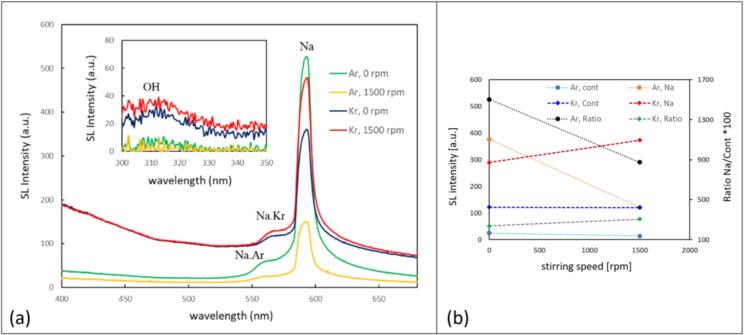
(a) SL emission spectra in the visible region and in the UV (inset) of the 3 M NaCl aqueous solutions in setup A at 21.2 kHz, with different stirring velocities for Ar and Kr, temperature 13 °C. (b) The changes in the sodium emission (589 nm in orange), the continuum (average from 450-480 nm in blue), and ratio sodium/continuum*100 (in black), with respect to the stirring velocities (0 and 1500  rpm), for Ar and Kr.

A similar picture as from the visual inspection of Fig. 4 arises from the spectra, see Fig. 5. Since we found an “optimum” stirring velocity for high Na*/continuum ratio in setup B around 1500 rpm, the spectral measurements in setup A were implemented at 0 and 1500 rpm for 3 M NaCl solutions saturated with Kr and Ar gas, respectively, and sonicated at 15 W. The spectra as depicted in Fig. 5a show again a broad continuum emission rising towards the blue, the Na* line at 589 nm together with the exciplex emissions (Na·Ar and Na·Kr around 554 nm and 570 nm, respectively), and here as well a small peak around 310 nm, attributed to OH*. Obviously, the Kr cases have higher continuum emissions and potentially somewhat higher intensities of the OH* in comparison to the Ar cases, attributed to the lower thermal conductivity and hotter collapse core temperatures. Thus, the hotter Kr bubbles emit more photons, and the higher temperature inside the collapsing bubbles leads to a higher amount of molecular dissociations and thus more OH* radical production.

Stirring leads to a striking reduction of the Na* peak for dissolved argon, together with a slight decrease in the continuum. In contrast, the Na* emission clearly increases in the case with dissolved krypton, while most of the continuum hardly changes. Expressed in the ratio of Na*/continuum emission, the opposing trends for the two gases are found again, see Fig. 5b black and grey lines. While this is, to our knowledge, the first report of such a startling difference between the SL emission features for the different gases under stirring, the Kr case at 7 °C (Fig. 4 right column) shows that these results remain sensitive to further parameter changes. Apparently, at the lower liquid temperature, influence by stirring on the colors is rather reduced, a strong orange emission being present already without stirring. Visually, the sodium and continuum emissions remain roughly similar. Still, the confining effect on the spatial distribution is discernible, i.e. the orange regions shrink somehow closer to the sonotrode (similar as discussed in Fig. 2a).

#### 3.1.3 Sonochemiluminescence

**Fig. 6:**
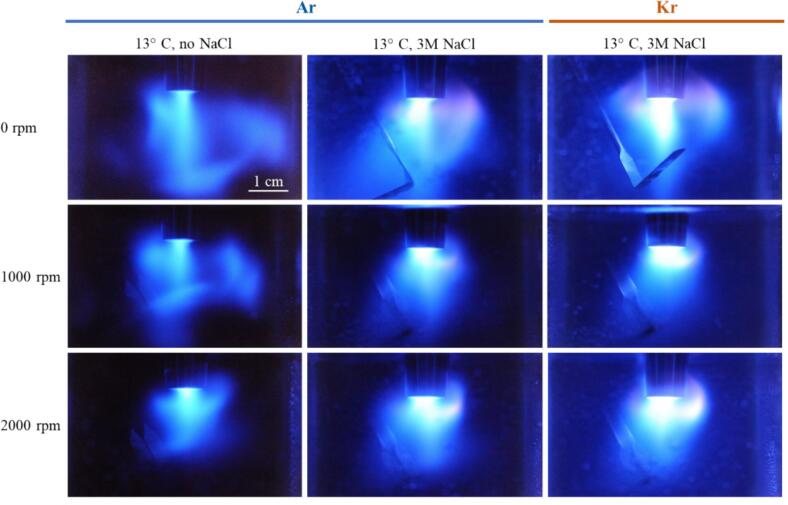
Spatial distribution of luminol SCL and Na* emissions in setup A at 21.2 kHz. Left column: DI water/ luminol purged with Ar. Middle column: 3 M NaCl aqueous luminol solutions purged with Ar. Right column: 3 M NaCl aqueous luminol solutions purged with Kr. Power was always set to 20  W, stirring rates are indicated at the different rows.

Sonochemiluminescence emissions of 0.1 mM luminol aqueous solutions are shown in Fig. 6. The cases with dissolved Ar without NaCl and with NaCl, as well as dissolved Kr with NaCl were investigated. In contrast to the native SL observations in Fig. 2, Fig. 4, the light emitting regions in the presence of luminol are larger and not confined to the sonotrode tip. This expansion of the luminescence region occurs probably owing to the fact that the threshold in the sound pressure field is lower for SCL than for native SL [10], [12]. Therefore, some cavitating bubbles that do not reach an SL activation temperature might still be activated for SCL. Further, some transport of OH radicals and/or excited luminol molecules by the liquid flow away from the tip might happen (see also the Supplementary Material and [66] on this point). In the presence of sodium, the Na* emission is readily recognizable, despite the extension and high intensity of the blue SCL emission. Sodium appears with rather violet color impression mainly to the sides of the tip. Native continuum emission cannot be perceived or separated from the blue SCL light, although the bright whitish emission below the tip might contain a part native SL continuum (see the Supplemetary Material for native SL without SCL). Adding NaCl visually seems to increase the overall intensity of emissions, in particular below the sonotrode tip. Indeed, a rudimentary color splitting by separating the RGB channels for some of the images of Fig. 6 shows a clear “red” part in the bright jet region for dissolved NaCl, being absent without the salt (see Supplemetary Material). As a main effect of stirring, a certain re-distribution of the emission zones is obvious in Fig. 6. More bubbles are confined to the sonotrode vicinity, and the long downward jet gets shortened and somehow bent with increasing stirring velocity for all three cases. Apparently the vortex flow created by the stirrer is cutting off and deviating the jet streaming flow from the tip (compare also Fig. 6D in Ref. [20] for a similar effect in a reactor with bottom transducer). Furthermore, stirring diminishes in all systems the overall light emission: The blue SCL regions are shrinking towards the sonotrode, and also the Na emissions are reduced in the salt solutions. The reduction effect is consistent with the tendency shown in Fig. 4 without luminol, columns 1 and 2 for Ar. In the experiments with luminol, for dissolved Kr stirring apparently does not lead to the intensification of sodium emission (like in Fig. 4 third column). Still, the visual impression confirms a certain persistence of the red color, contrary to Ar.

**Fig. 7:**
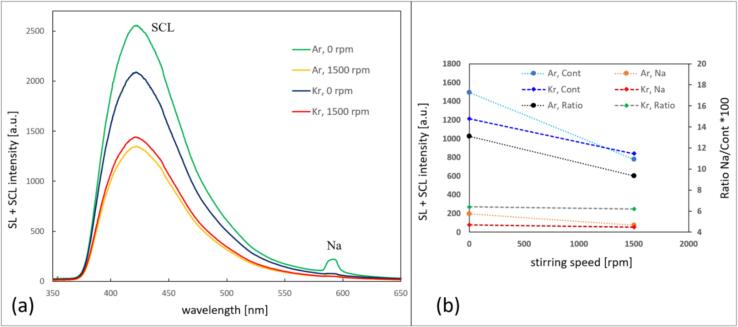
(a) Emission spectra of 0.1 mM luminol 3 M NaCl aqueous solution in setup A at 21.2 kHz for Ar and Kr, stirring velocities indicated. Note that very small signals of Na* are present in all spectra. (b) Changes in the sodium emission (around 589 nm, in orange/red), the continuum (average from 450-480 nm, in light/dark blue), and the ratio sodium/continuum*100 (in black/green), with respect to the stirring velocities (0 and 1500  rpm), for Ar and Kr. Here, “continuum” naturally contains both SCL emission and native SL. Within the “continuum” window, SCL intensity should be roughly 3-fold SL intensity (see Supplemetary Material).

Fig. 7 a and b show the spectral data of the 3 M NaCl aqueous solutions for Ar and Kr, suggesting that although stirring affects the SCL significantly, the amount of this reduction is different for Ar and Kr. No emission is observed below approximately 370 nm because of light absorption by luminol. While a clear Na* signal appears in the Ar case, which is also clearly reduced by stirring, the Na* line occurs only very weakly in the spectrum obtained with Kr, but it also seems diminished under stirring. Interestingly, the SCL emission from the spectral data with Ar appears somewhat brighter than with Kr, although Fig. 6 gives a different visual impression. As well, the collapse core should be hotter for the latter gas, and we saw a stronger native SL signal in Fig. 4, Fig. 5 above. Also OH / H_2_O_2_ production should be higher under Kr in pure water [67], [68], which is supported by calculations of mixture segregation of water vapor and noble gas [69]. Potentially, the influence of the salt might lead to deviations from this expected behavior, but this needs closer investigation (compare [50]). Therefore, we assume that this specific outcome is due to trial-to-trial variability, estimated to amount to about 20 %. It should also be kept in mind that emission spectra do not represent a global measurement, i.e., correspond only to a limited emitting zone.

With respect to the interpretation of the ratios of sodium to continuum emissions in Fig. 7b, caution is necessary, since the continuum includes both native SL and luminol SCL. While a disentanglement of the respective contributions is cumbersome and beyond the scope of this work, we show in the Supplemetary Material that at 359  kHz a relative intensity of SCL/SL ≈ 3 is found for 0.1  mM luminol concentration. One might assume that this translates more or less to the lower frequency case of 21  kHz here. However, since the luminol molecule might enter into non-spherical bubbles, it might be questionable anyway if SL continuum and Na* emissions remain totally unaffected, and thus if SL (without luminol) could just be subtracted from SL + SCL (with luminol) to find the clean SCL contribution.

### 3.2 Ethylene glycol

For ethylene glycol / NaCl solutions, again the effects of stirring on the SL emission locations and emission spectra were studied with dissolved Ar and Kr gas. Further, sonochemiluminescence was investigated. An important parameter change in comparison to water is the higher viscosity.

#### 3.2.1 Stirring and noble gas effects

**Fig. 8:**
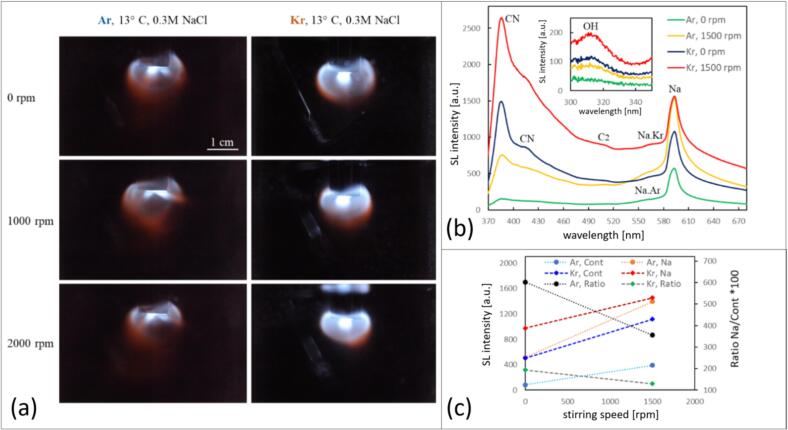
(a) Spatial distribution of the emission in 0.3 M NaCl EG solution sparged with Ar (left) and Kr (right) in setup A at 21.2 kHz, 20 W (exposure time 5  min). (b) Emission for Ar and Kr without and with stirring at 1500  rpm. Annotations for CN and C2 are tentative. (c) Changes for Ar and Kr in the sodium emission (around 589 nm, in orange/red), the continuum (average from 450-480 nm, in light/dark blue), and the ratio sodium/continuum*100 (in black/green), with respect to the stirring velocities (0 and 1500  rpm).

As it can be seen in Fig. 8, the spatial SL patterns in EG are somehow different from the water case (Fig. 4). They rather resemble the colored SL patterns reported under sonotrodes for noble gases in concentrated sulfuric acid with Na_2_SO_4_ [28], [32]: Below the horn tip, a blue-white bulb appears with orange extensions to the bottom. Inside the bulb, a bright white jet (Kr) or double jet (Ar) can be seen which appears centrally at the tip. In the aqueous NaCl solution (compare Fig. 4), the bulb around the tip appears orange and not blue, and the central bright white region forms rather a cone and covers the full tip area (which possibly might be expected in EG as well, but for higher driving power, as reported for sulfuric acid by Xu *et al.* [28]). Also Choi reports a transition from bulbous to conical blue emission below a horn in 68 % glycerine without dissolved sodium salt [31].

Comparing the two noble gases shows a greater overall emission of Kr together with a more extended blueish region, whereas the orange emission in Ar reaches more towards the tip. The spectra in Fig. 8b show that although a considerable part of the emissions in the UV region will be lost due to absorption by the liquid and the PMMA container, EG has clear emissions in the UV (inset), where the OH radical at 310 nm can be identified. This feature is more pronounced than in water (Fig. 5a, inset). Again the light emission by OH* radicals is more intense for Kr. Further molecular peaks are discernible as shoulders: Na·Ar and Na·Kr exciplexes (∼560–570 nm) next to the Na-D line (589 nm), two peaks (385 nm and 415 nm) that may be attributed to CN, and one (512  nm) that may indicate the presence of Swan bands. Apart from water splitting, the OH radical emission possibly could as well originate from the dissociation of hydroxy groups of EG, and its intensity is higher by a factor of about 5 than for the DI solutions. CN emits prominently, and it is known as a very strong emitter in other SL systems [70]. CN emission can arise in the presence of nitrogen from air traces, which is likely due to the high viscosity of the solution.

Contrary to the water case, in EG the SL emission shape as seen by the eye is hardly affected by stirring. A closer inspection and the spectra in Fig. 8 reveal, however, a clear intensification of the emissions under stirring for both Ar and Kr. The visual impression shows a certain extension of the orange regions for Ar and some increase in brightness of the blue bulb for Kr. In the spectra we see for both gases a stronger increase in the continuum than in the Na* line, leading to a decrease of the Na/cont ratio.

#### 3.2.2 Sonochemiluminescence

**Fig. 9:**
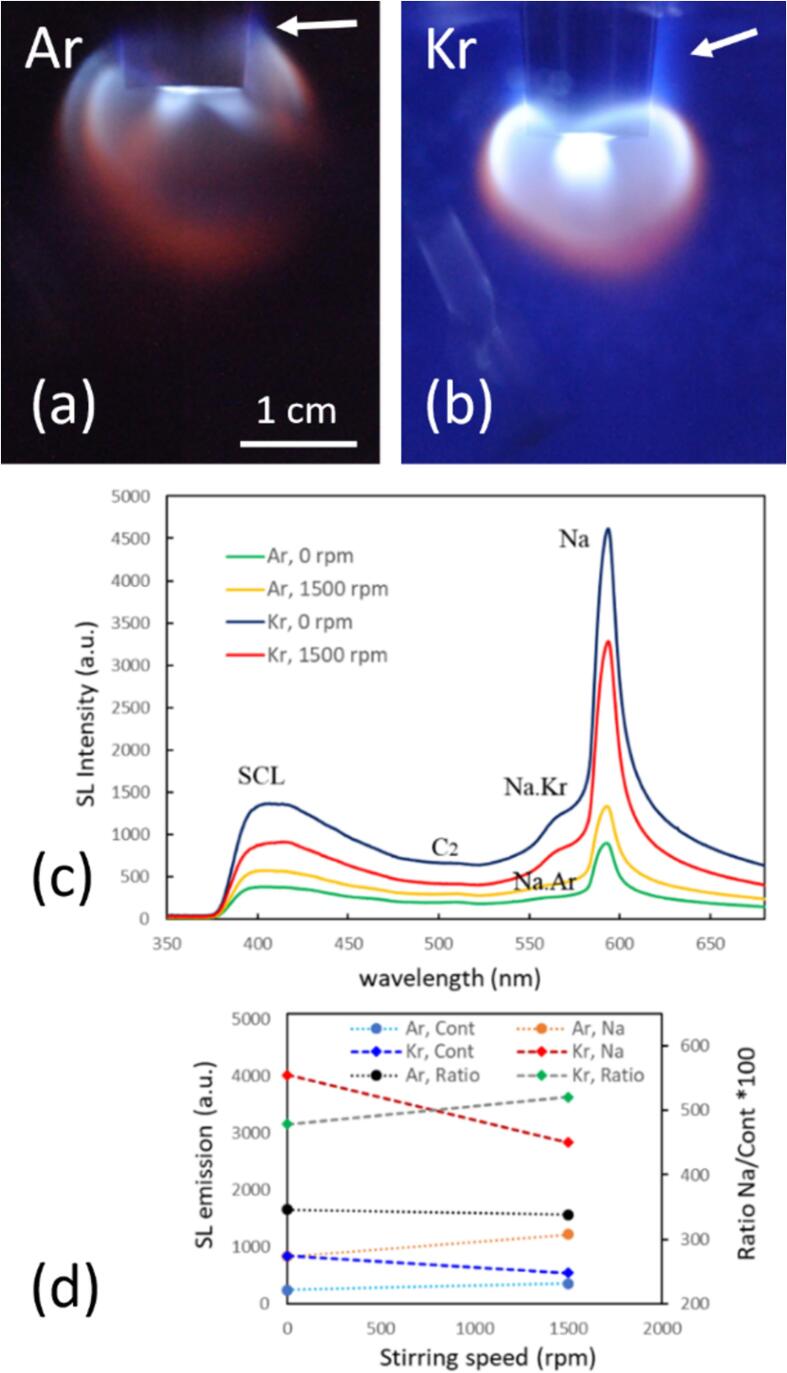
Emissions in sonicated 0.3 M NaCl 0.1 mM luminol EG solution, setup A, 21.2 kHz, 20  W, scale indicated. (a), (b): Spatial distribution for Ar (Kr) saturated solution, stirring at 1000  rpm, 5  min exposure. The arrows indicate apparent “pure” luminol emission regions. (c) Optical emission spectra for both gases and runs without stirring and with stirring at 1500 rpm; emission features indicated, annotation for C2 is tentative. (d) Changes in the sodium emission (around 589 nm, in orange/red), the continuum (average from 450-480 nm, in light/dark blue), and the ratio sodium/continuum*100 (in black/green), with respect to the stirring velocities (0 and 1500  rpm), for Ar and Kr. Here, “continuum” naturally contains both SCL emission and native SL.

Spatial emission patterns and the spectral data of the 0.1 mM luminol 0.3 M NaCl EG solutions sparged with Ar and Kr and stirred at 1500 rpm are shown in Fig. 9. Since stirring apparently had only a small effect on the spectra and did not change significantly the camera images, we just present the visual impression for medium strong agitation. The emission shapes are nearly identical to the case without luminol (Fig. 8), but now a more or less homogeneous blue emission from the bulk liquid appears additionally, that is quite pronounced for Kr. Furthermore, there are regions at the side of the sonotrode shaft where apparently predominant luminol emission appears: A brilliant blue color, distinct to the whitish-blue color of the native SL, occurs from a zone that was absent of detectable SL emissions without luminol (Fig. 8). This possibly “pure luminol” region seems to form a ring around the lower sonotrode shaft, and it extends more to the upper part of the rod for Kr. However, we do not have an explanation yet of the prevalent chemical activity within this region. Comparison with Fig. 6 for SCL in the aqueous case shows that a certain activity at the shaft appears as well, but it is more diffuse to the region below the tip. See also the Supplementary Material, Figs. S4 and S5, for a rudimentary spectral analysis of the emission zones of Fig. 9a using the picture’s RGB channels. We note that SL and SCL emissions from the side of the sonotrode have been reported before, essentially due to bending or thickness modes of the horn; see for instance Bampouli et al. [71]. For poly-ethylene glycol / water mixtures just (and only) in a viscosity range comparable to EG (between 6 and 22 cP), these authors find emissions at the lower shaft, quite similar to the “pure luminol” regions in Fig. 9a. This emission region seems to be connected with the peculiar (hemi)spherical bubble structure around the tip, and not necessarily with further horn modes. We conclude this from their images of bubble structures at different viscosities, see their Fig. 5 in [71].

Comparing the spectra in EG / NaCl without and with luminol (Fig. 8, Fig. 9), one notices that the former UV part is totally absorbed by the luminol. Further, the large CN peaks are not seen in the run with luminol, potentially because less air remnants have been present.

The other spectral features (C_2_, Na·Ar / Na·Kr and Na*) remain as without luminol. The absolute emissions in the Ar case with luminol have shown roughly the same brightness as without, but in the Kr case, the emissions increased significantly after luminol addition (note the different scales in the spectra of Fig. 8 and Fig. 9).

A further comparison of luminol in EG (Fig. 9) and luminol in water (Fig. 6, Fig. 7) shows that the luminol SCL emission much more dominates over the native SL in the aqueous case. Additional features (C_2_, Na·Ar / Na·Kr) are visible in EG but are nearly absent (or masked by luminol) in water. One reason behind this might be the low share of water in the EG, supposing that the luminol excitation involves OH radicals and OH^–^ ions, similarly to the aqueous solutions case.

### 3.3 Phosphoric acid

The experiments reported in the following were done in setup B. Phosphoric acid (85 %) with dissolved sodium phosphate and sparged with argon, krypton, or xenon was sonicated at 36.5  kHz and subjected to stirring.

#### 3.3.1 Sonoluminescence

**Fig. 10:**
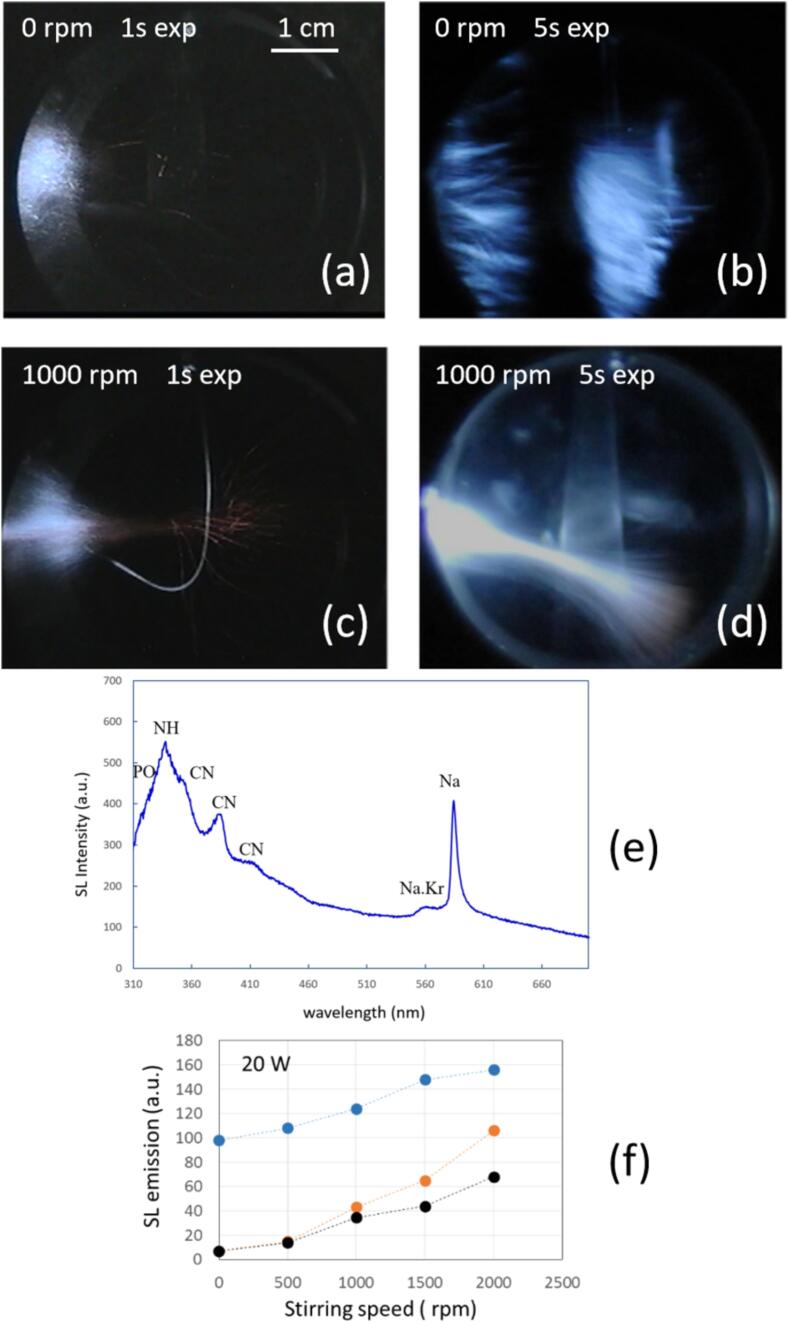
Sonoluminescence in 85 % phosphoric acid, 0.5 M sodium phosphate, sparged with krypton. Setup B, 36.5 kHz, 20 W, scale indicated, transducer to the left. (a), (b): SL without stirring, 1  s and 5  s exposure time, respectively. (c), (d): SL immediately after onset of stirring, exposures 1  s and 5  s. (e) SL emission spectrum at 2000  rpm, showing continuum, sodium and additional lines (see labels). (f) Changes in the sodium emission (589 nm – orange), the continuum (average from 450-480 nm in blue), and the ratio of sodium/continuum*100 (in black) with respect to the stirring velocities (0–2000  rpm).

Fig. 10 shows typical long-term exposures, a typical spectrum with stirring and Na* emission line-to-continuum ratios of the system. Characteristic tracks or streaks of emission appear, each of which potentially corresponds to the path of an individual bubble moving away from the transducer (from left to right; compare the bubble tracks shown in [32]). Stirring increases the light output, apparently by essentially multiplying the number of bright streaks. Additionally, the streaks concentrate in a cone-like fashion, reminiscent of structures under a larger sonotrode emitter [72], [73]. As can be seen in the 5  s exposures (Fig. 10b and d), initially SL emissions originate from two (possibly antinodal) regions, one in front of the emitter and the second opposite to it, similar to some images in water (Fig. 2). Orange emissions are rather faint, but well discernible for the stirred system. Stronger stirring destructs the second (far) antinodal emission and enhances the SL emission from the primary antinode, similar to the aqueous case shown in Fig. 1b. At the same time, it leads to the very bright bubbles in the vicinity of the transducer and the orange emission at the tail. The spectrum in Fig. 10e reveals that apart from a broad continuum and the sodium peak with a Na·Kr shoulder, also larger features in the range of 300 – 400 nm appear. These are contributed by NH and CN, and possibly PO*. We refer to them below. Analysis of the stirring induced spectral changes are reported here first for the ratio of sodium to continuum (Fig. 10f). It shows that both line emission and continuum emission are rising with stirring speed, but the sodium emission grows at a higher rate. Thus the ratio of sodium to continuum rises as well, with a maximum achieved at the highest stirring velocity. This coincides with the visual impression from Fig. 10b and 10d and the findings in aqueous solutions in setup B where stirring induced a shift towards orange emission. Differently to NaCl in water, no maximum at 1500  rpm is found in the acid for the investigated parameters.

**Fig. 11:**
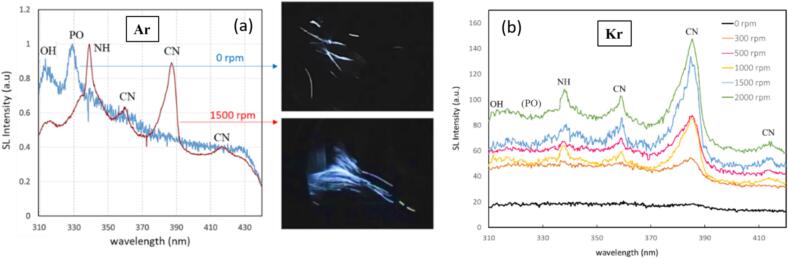
SL emission spectra of the 85 % phosphoric acid / 0.1 M Na_3_PO_4_ solution in the UV region at different stirring rates and fixed acoustic power of 20 W), setup B. (a) Ar flow: spectra for 0 rpm (blue) and 1500 rpm (red) are shown, normalized to the maximum (overall light emission at 0  rpm being weaker by a factor of about 8). Photographs (5  s exposure) are related by arrows; transducer to the left. (b) Kr flow: spectra for increased stirring speeds, not normalized.

Fig. 11 presents the slightly extended UV region of the SL emission spectra of the phosphoric acid solution for different stirring velocities, once for Ar and once for Kr. This part of the spectra reveals a peculiar result as several components of the spectrum become prominent peaks with stirring. Typical peaks expected for SL emission from phosphoric acid are OH and PO* at 310 nm and 325 nm, respectively. They are usually attributed to different bubble populations, OH from spherical and PO* from drop-injecting bubbles, since the latter comes from pyrolysis of the non-volatile H_3_PO_4_ [74]. Here we see OH and PO* clearly for Ar without stirring (Fig. 11a), but for Kr without stirring, no clear features arise over the continuum (Fig. 11b).

Under stirring, in the Ar case the continuum is strongly raised. However, for comparison of the spectral signatures, the non-stirred curve in Fig. 11a has been vertically expanded by a factor of about 8, i.e., the curve maxima have been equalized (visible by the larger noise levels in the trace). The PO* feature disappears under stirring, or is overlaid. OH persists, and strong NH at 335 nm and CN components at 360, 385 and 415 nm appear additionally. Similarly to the EG case, CN emission should indicate the presence of dissolved air in the liquid, the intrabubble reaction of nitrogen and CO_2_ being known to lead to CN emission [70]. This can even hold in the case of remaining dissolved air with its rather low CO_2_ content, i.e., for partial degassing (see the example in Fig. S8, Supplementary Material). Thus the stirring seems to activate and/or introduce air remnants in the acid, which can explain the lines. However, the overall increase in brightness is also very likely accompanied by a noticeable change in multi-bubble dynamics, as shown below. For stirring in the Kr case, the continuum is as well strongly increased, OH is raised, and the same apparently “parasitic” lines from air occur. PO* does not clearly appear and remains absent or hidden (compare as well the spectrum in Fig. 10e without clear PO*). The photographs in Fig. 11 show again the emission along streaks, i.e. presumably individual bubble paths, that multiply under stirring.

It is tempting to suppose the origin of both Na* and PO* from the same bubble population, namely bubbles with some form of droplet injection, because both components should not be in the vapor. Therefore, one might expect a simultaneous emission of both lines. However, here we cannot confirm a coincidence of Na* and PO* emission, and while Na* rises with stirring, PO* even vanishes for Ar where it occurred without stirring. Possibly further effects come into play, for instance a quenching of PO* by the introduced air [75]. Thus the link of PO* and Na* to the according bubble population(s) would need further study at this point.

**Fig. 12:**
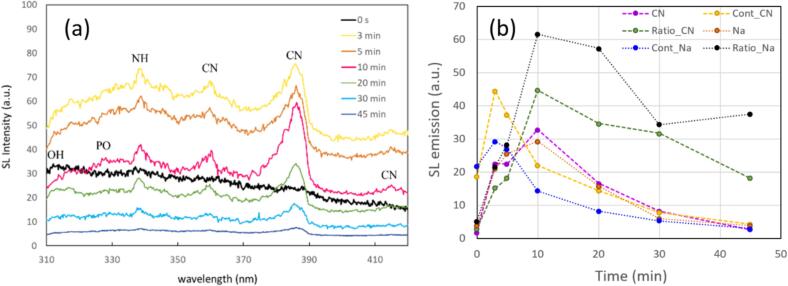
Time dependence of SL emission in PA under the stirring velocity of 1000 rpm. Other conditions fixed at 36.5 kHz, 20 W of acoustic power and 60 ml/min of Krypton flow, setup B. (a) UV part of the spectra, duration of stirring indicated by color. All spectra to the same scale. (b) Temporal variation of sodium emission (589 nm – orange), the continuum (average from 450-480 nm in blue), and the ratio of sodium/continuum*30 (in black) with respect to time of applied stirring. Additionally shown is the evolution over time of the CN peak at 385  nm over continuum (pink), the continuum between 395–405  nm (yellow), and the ratio of CN and this continuum value times 30 (green).

Since apparently remnant or re-gassed air plays a role in the SL emissions, we probed the spectral evolution over a longer time interval of 45  min. Indeed, the observed SL spectra vary in total emission intensity and in line strength, as is shown in Fig. 12. On the scale of several minutes after switching on the stirrer, the overall intensity strongly rises, but then falls even below the non-stirring values (Fig. 12a). However, most lines still persist over up to 30 min of stirring, even when the overall intensity has dropped below the starting level. While the continuum starts to fall already after about 3  min, the lines and their contrast relative to the continuum rise further until about 10  min. This is depicted in Fig. 12b (note that two continuum levels have been extracted, both essentially coincident). Interestingly, Na* and CN(385) run concordantly with their maximum after 10  min, clearly shifted relatively to the continuum. OH and PO* emissions do not show sufficient contrast to be evaluated fully, but apparently a weak OH component rides always on the continuum. PO* shows up just at the maximum line contrast after 10  min of stirring, which might be interpreted as some correlation with Na*, but this is not conclusive.

The considerable increase in SL intensity after starting stirring seems to a large part to be caused by an increased number of strongly emitting bubbles, potentially due to some redistribution of nuclei and/or change in bubble dynamics. At the same time, CN and NH line emissions are stimulated, contributing to the enhancement and pointing to an increased dissolved air concentration in the acid. Reactions of CO_2_ and N_2_ in the bubbles might later, at higher air concentrations, “poison” the plasma and lead to an overall reduction of SL light. Apart from air, diffusional or convectional longer-term effects should as well be related to the sparged noble gas (here Kr). A certain re-distribution of Kr in the container might account for an increase in brightness, while later outgassing could decrease SL emission. This would mean that in spite of noble gas sparging the overall level of dissolved Kr decreases in the long run, adjusting to a rather low level under continuous sonication. Possibly other sparging techniques (e.g. via multiple tubes) might avoid this.

Although liquid temperature (which was not rigorously controlled within the indicated measurement) might have some additional influence, we finally conclude that the variations of the emission spectra are to a good part due to the dissolved gas content, which is affected via stirring of liquid open to the atmosphere. Still, the effect of mechanical agitation on the bubble dynamics and the bubble populations remains to be clarified, potentially as well coupled to the gas content, of course. A rudimentary spatial analysis of the emission spectra via RGB channel splitting of SL images leads to the conclusion that no strong separation of blue and red occurs, suggesting that bubbles in the streak do emit significant continuum and/or lines in the blue (see Supplementary Material, Fig. S6). Thus the same bubbles might produce all lines, coherent with their coincidence under temporal evolution. While a comprehensive assessment of the detailed bubble behavior in stirred cavitating systems is beyond the scope of this article, we would still like to present some observations by high-speed recordings in phosphoric acid in the following.

#### 3.3.2 Bubble dynamics

In the H_3_PO_4_ system of setup B, we recorded high-speed movies of the sonoluminescing cavitation streamers without and with the effect of stirring. Here, the acid was saturated with xenon to allow for ultra-bright SL, being observable simultaneously with the bubble dynamics. The visual impression under Xe was similar to the cases of Kr and Ar, and we suppose that the main mechanisms of SL enhancement under stirring are the same for all three gases. For a detailed description of the method and the setup we refer to [76]. In short, illumination and camera settings were chosen such that shadowgraph imaging of the bubbles was just possible, but SL flashes still had contrast on the camera sensor.

**Fig. 13:**
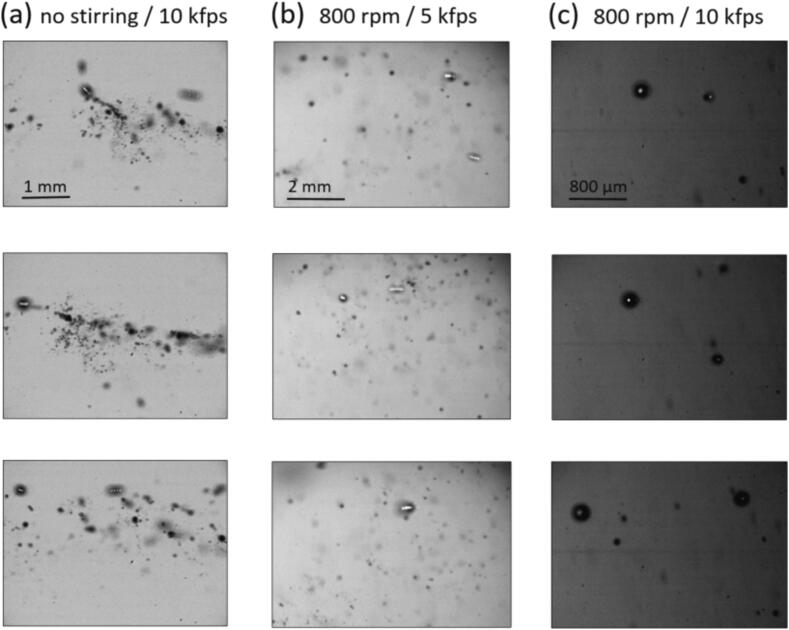
Frames from high-speed movies recorded in Xe-saturated H_3_PO_4_ in setup B at 12 W and 36.5 kHz. a) Without stirring, 10,000 fps, exposure time 100 µs. b) With stirring at 800 rpm, 5000 fps, exposure time 200 µs. c) With stirring at 800 rpm, 10,000 fps, exposure time 100 µs. Note that the transducer is here to the right, and main bubble motion is from right to left. Several subsequent SL flashes of large moving bubbles can be seen as bright dots in the bubble center due to the exposure time being longer than the acoustic period.

Stirring definitely produces a noticeable effect on the visual appearance of the bubble field. Fig. 13 shows several frames selected as examples of cavitation within bubble streamers recorded inside the cone-shaped region in front of the transducer, which is positioned here to the right. Bubbles mainly move to the left, i.e. away from the transducer surface. The typical appearance without stirring, column a) in Fig. 13, shows that individual bigger bubbles with SL activity are accompanied by a trail of smaller bubbles in their wake. This swarm of small and apparently non-luminescing bubbles has a significant influence on the bigger bubble’s motion, resulting in frequent changes of its trajectory and speed. A different situation is recorded inside the conically shaped streamer region formed under stirring. SL active bubbles run away from the transducer, but the entourage of smaller bubbles is missing. In terms of bubble dynamics, the cluster behavior that linked bigger, frequently emitting bubbles and the smaller (apparently not emitting) bubbles is no longer prevalent – the larger bubbles run more “free”. In general terms, under stirring in acid the clustering and coalescence between bubbles of the smaller sizes appear reduced. However, the coalescence of big, sonoluminescing bubbles seems to be facilitated due to the absence of the swarms of smaller bubbles, which leads to even larger, rather isolated, moving bubbles. In effect, under stirring conditions, we recorded the largest individual sonoluminescing bubbles in phosphoric acid, which at the same time showed the brightest SL flashes. See also the sample movies in the Supplementary Material.

## 4 Discussion

In the following, the variety of observations and some explanations are briefly summarized and discussed.

### 4.1 Sonoluminescence patterns

The observed characteristic spatial appearance of colored SL emissions in setup A in a bulbous form is essentially in accord with previous reports in acids for medium high powers [28], [58], [32]. Here we observe a similar pattern also in aqueous and ethylene glycol NaCl solutions: Under the sonotrode, the SL pattern consists of an attached or close region at the tip with bright white emission. Farther from the tip and sometimes surrounding it, a type of (hemi)spherical or bulbous region occurs which typically shows white-blue color in the center and orange emissions farther out. Additionally, a jet can emerge from the sonotrode tip emitting bright white light, but potentially changing its color to orange at some distance, in particular when leaving the hemispherical region (we note that Choi [31] reported a fully orange region below the sonotrode tip in 1 M aqueous NaCl solution). The overall cavitation bubble structure might be explained by acoustic forces in the decaying acoustic field near the tip, where medium sized bubbles can gather on an equilibrium positions on a nearly spherical shell ([77], see also [78]). Details of the pattern, however, should depend on acoustic pressure, liquid properties, and involved bubble sizes, which might be the reason why it has not been reported yet for aqueous systems. Higher liquid viscosity seems favorable for the structure’s development, potentially because of effectively larger cavitation bubbles that find their equilibrium position at a lower pressure amplitude and thus at a larger distance from the tip.

In setup B, which rather develops a standing acoustic wave, the patterns in aqueous NaCl solution are again similar to previous reports [30], [31], [50]: blue-white emissions near the transducer and antinodal regions, and orange between antinodes and nodes. In phosphoric acid, the pattern remains, although orange emissions seem less pronounced than in water and appear more confined to the rapid flow directed away from the transducer plate.

### 4.2 Sonochemiluminescence patterns

The SCL mechanism is mediated via OH radical production and its transfer to the liquid phase, and the SCL activation threshold in terms of sound pressure has been reported to be typically lower than that for native SL [10]. Thus SCL generating bubble populations and accordingly light emission patterns are expected to differ from SL. SCL patterns have only been recorded in setup A, and the observed characteristic spatial distribution of SCL agrees with many previous reports of SCL below a sonotrode horn (e.g. [79]), namely a cloudy zone that seems to be transported away from the tip by the jet flow and further fluid convection. Here we have shown that in the presence of Na, additionally orange zones can be resolved near the sonotrode tip sides, just like in the cases without luminol. With respect to SCL in EG, we note that cavitation regions of distinctive SCL activity can be seen at the sides of the sonotrode tip. The exact chemical pathway is difficult to assess, and we have not found previous reports on SCL in EG. While OH might be split from EG (facilitating SCL), the water fraction of the EG solution – although small – should play a crucial role in mediating the luminol emission (and thus might be limiting the process). The details would need further investigation.

### 4.3 Overall stirring effects

Since the stirring impeller head speeds $v_{h}$ remain well below sound speed, no *direct* impact on the ultrasound propagation in the cuvette should occur by stirring, i.e., no significant stretching or compression of the acoustic wavelength. However, significant influence of stirring on liquid flow and bubble distributions should be expected if $v_{h}$ reaches or overcomes typical acoustic streaming flow speeds $v_{\mathrm{stream}}$ of the system and/or typical bubble translation speeds $v_{\mathrm{bub}}$, i.e. $v_{h}\approx v_{\mathrm{stream}}$, $v_{h}\approx v_{\mathrm{bub}}$. With $v_{h}=0\cdots 4$ m/s, $v_{\mathrm{stream}}\approx 1$ m/s in the sonotrode jet flow, and $v_{\mathrm{bub}}\approx 1$ m/s due to primary Bjerknes forces, both conditions are fulfilled. Thus the induced flow increases liquid transport which includes transport of solid or gaseous nuclei and of small bubble remnants. These are supposed to behave like passive tracers following the liquid as long as acoustic forces on them remain small, i.e. their size stays below the local activation (Blake) threshold value. Since the sound pressure amplitude and therefore the Blake threshold is generally a function of spatial position in the acoustic wave, stirring is expected to shuffle more nuclei into high pressure zones, crossing their activation threshold and thus increasing the active cavitation bubble population. This is of particular importance at higher power when pressure antinodes can become repulsive for small bubbles due to Bjerknes force reversal [80] and show reduced or no SL activity. Indeed, high bubble density under stirring in otherwise void pressure antinodes has, for instance, been observed by Hatanaka *et al.* [26].

Stirring, as applied here in setup A, changes the global flow field by interaction of the impeller vortex with the sonotrode induced jet flow. A higher overall cavitation activity due to redistribution of nuclei by stirring is most probably given in our experiments, but we do not see coherent trends in all the data. SL emission has been observed to go up under stirring in most systems, but also to drop in other cases (notably for the continuum in setup B with Kr in aqueous NaCl solution, and for the overall emission in setup A in aqueous NaCl solution with Ar). Besides, an increased light emission does not necessarily relate to more active cavitation bubbles, but might also be due to larger and more intensely emitting bubbles (like probably in the PA case in setup B).

Stirring of the aqueous luminol systems in setup A clearly shows effects from the large scale liquid convection: the emissive jet region below the tip is bent to the side, and the cloudy emission regions are confined closer to the tip, which actually reduces the overall emissions. Stirring effects on SL and SCL in the more viscous EG (setup A) remain small, possibly because of the high viscosity and accordingly an impeller Reynolds number at the edge to laminar flow. However, in PA (setup B), which has as well higher viscosity, the effects are larger and the overall emission is enhanced. The difference might stem from the different geometry, particularly the relative positions of transducer and impeller.

As outlined above, the spatial separation of SL spectral components in all systems is usually attributed to different bubble populations, the orange emitting bubbles being injecting liquid while the white-blue ones remain essentially spherical without spraying liquid inside. Here we have shown that mechanical stirring can affect both these bubble populations, leading to changes in the color patterns. Among the multiple effects induced by stirring, one main impact in our setups apparently was an increase of gas diffusion, both within the liquid and via the free liquid surface. Both are likely mediated by an increased turbulent shear by the agitation. PA solution, initially saturated with noble gas, showed under stirring spectral lines including CN and NH raising over minutes, indicating air in the collapsing bubbles. Thus we conclude that stirring can “activate” dissolved rest gas by fostering turbulence and gas diffusion towards the cavitation bubbles, as well as increase re-aeration of the liquid with air from the atmosphere. At the same time, out-gassing of the noble gas is suggested by a reduction of the continuum and later of the emission lines over a time scale of tens of minutes. Any connection of gas diffusion to different active bubble populations still needs more exploration.

### 4.4 Bubble de-clustering

In contrast to passive nuclei, active (i.e. strongly oscillating) bubbles show a considerable motion relative to the liquid, caused by primary and secondary Bjerknes forces [6], and we have seen that $v_{h}\approx v_{\mathrm{bub}}\approx 1$ m/s. Previous interpretations of the effects of stirring on SL, SCL, and sonochemical yields [26], [20] suggest that coalescence of small bubbles is hindered by the flow, leading to the observed effects (e.g. enhancement of SL and SCL at high acoustic power by stirring). This would imply that the enhanced bulk liquid motion disturbs mutual attraction of active bubbles. Here we share this idea, and we would like to add more details of a potential underlying mechanism. In particular, we consider ensembles consisting of a larger bubble and a group of smaller bubbles nearby. This seems to be a generic pattern (although not the only one) found in streamers, double layer structures, and clusters (see [81]). Here we observe it in non-stirred PA streamers (Fig. 13a). Under stirring, a separation of large and small bubbles takes place, i.e., these bubble populations are apparently affected differently. We hypothesize that a cross-flow mechanism can contribute to this separation inside a streamer, i.e. if all bubbles are migrating unidirectionally under the action of the primary Bjerknes force in the non-disturbed case. Superimposed side flow induced by stirring could be able to “wash away” smaller bubbles from the track of moving larger ones, as observed in the movies. This appears feasible since the primary Bjerknes force essentially scales with the bubble volume (i.e. $R_{b}^{3}$ with the time averaged bubble radius $R_{b}$), while the viscous drag force mediated by the liquid flow relative to the bubble scales basically with the bubble projected area (i.e. $R_{b}^{2}$) at higher Reynolds numbers. For low Re (Stokes flow regime) the drag scales even linearly with $R_{b}$. Thus the ratio of primary Bjerknes to drag force is expected to rise with the bubble radius as $R_{b}$…$R_{b}^{2}$, which shows that the smaller bubbles are more affected by cross-flow than the larger ones. The effect should become more pronounced in higher viscosity liquids, since Reynolds numbers are accordingly lower, which could explain a direct observation of de-clustering in PA with its about 40-fold higher viscosity than water. For the color distribution in PA, this might lead to a relative increase of orange Na* emissions vs. continuum, since the larger bubbles which are more prone to droplet injection are less affected and less shielded by smaller ones. Thus they can oscillate stronger and move faster, which supports both SL and droplet injection via translation induced jetting [55], [32]. Whether such a mechanism could be viable also for aqueous solutions would need further evaluation of the corresponding bubble dynamics, which is under way. Also, the effect of increasing acoustic driving pressure on the de-clustering might be explored. One would expect that a stronger driving leads again to clustering [26] due to the raise of secondary Bjerknes forces [82], [83] in comparison to crossflow drag forces. This should quench SL and appear similar to the unstirred case.

### 4.5 Noble gas effect

Generally, SL should get brighter with lower heat conductivity of the gas in the bubble [3], and accordingly we see in most cases an intensification of SL if we switch from Ar to Kr to Xe (Xe only in PA). However, in our experiments this refers mainly to the blue/white emission regions that become brighter and more extended. The orange Na* regions can also shrink or remain roughly the same under Kr as compared to Ar (Fig. 4). Most remarkable, the stirring effects partly differ for dissolved Ar and Kr in an opposed way. Liquid agitation of aqueous NaCl solution enhances Na* for Kr in setup A and setup B, while it quenches Na* for Ar in setup A. The quenching also appears under Ar in luminol solution, whereas stirring with Kr in luminol solution appears not to quench Na* emission. Enhancement of the Na* line under stirring similarly appears for Kr in PA (unfortunately, we have no data on Ar in PA). Supposing any type of non-spherical or disturbed bubble shape for the sodium line to appear, it seems apparent that stirring contributes to such disturbances by imposing additional asymmetries to the bubble environment (relative fluid motion and shear forces). Therefore, an enhancement of droplet injection and orange emission should be expected. However, since the influence of stirring is manifold, more aspects might come into play. Bubble de-clustering and/or enhanced gas diffusion could change bubble sizes and bubble contents, leading indirectly to a changed dynamics. In this context, different solubilities of the gases in the liquids could play a role, but this has to remain speculative here.

## 5 Conclusion

Stirring sonicated liquids opens up further degrees of freedom in processing parameters for sonochemical systems. However, mechanical agitation is non-trivially coupled with a variety of physical effects, thus complicating a clear strategy for its employment. Here we have shown that SL and SCL spatial distributions and spectral properties can be affected and changed in aqueous and more viscous systems by moderate stirring (up to 2000 rpm). In essence, mostly an enhancement of sodium emission and a variation of continuum emission was found for increased stirring. As important underlying effects, enhanced gas diffusion (probably by increased turbulence) and bubble de-clustering (possibly by differential drag between different bubble populations, leading to their separation in cross-flow) have been identified. In the specific example of phosphoric acid, stirring induced de-clustering leads to large isolated bubbles with extremely bright sonoluminescence flashes. Further observed stirring effects relate to changes in macroscopic flow patterns and redistribution of nuclei and chemical components in the reactor. Although certain trends prevail in our results (e.g. an overall increase of emission, as well as a strong enhancement of Na* line emission), they are not necessarily robust under variation of further parameters like dissolved gas, acoustic field or liquid properties. This finding is in accord with previous reports on sonochemical yields under liquid agitation (see [20], [21] and references therein). In conclusion, its multiparameter sensitivity presents stirring as a potentially useful and valuable tool for sonochemistry, where a large benefit might be achieved with low effort. On the other side, care and measures should be taken to really affect the desired physical factors and finally reach a higher sonochemical activity. Additional research and more detailed studies might be indicated to further develop stirring as a successful tool.

## CRediT authorship contribution statement

**Atiyeh Aghelmaleki:** Writing – review & editing, Writing – original draft, Visualization, Investigation, Formal analysis, Data curation, Conceptualization. **Hossein Afarideh:** Writing – review & editing, Supervision, Methodology, Funding acquisition, Formal analysis, Conceptualization. **Carlos Cairós:** Writing – review & editing, Writing – original draft, Visualization, Investigation, Data curation, Conceptualization. **Rachel Pflieger:** Writing – review & editing, Validation, Methodology, Formal analysis. **Robert Mettin:** Writing – review & editing, Supervision, Methodology, Funding acquisition, Formal analysis, Conceptualization.

## Declaration of competing interest

The authors declare that they have no known competing financial interests or personal relationships that could have appeared to influence the work reported in this paper.

## Data Availability

Data will be made available on request.
