# Supplementary material for: Effect of mechanical stirring on sonoluminescence and sonochemiluminescence
Source: Ultrason Sonochem. 2024 Nov 6;111:107145. doi: 10.1016/j.ultsonch.2024.107145 (PMC11615137; doi:10.1016/j.ultsonch.2024.107145)
Supplement: SpectralAnalysis.pdf [file mmc4.pdf]

Supplementary Material

for the article

"Effect of mechanical stirring on sonoluminescence and sonochemiluminescence"

by A. Aghelmaleki, H. Afarideh, C. Cairós, R. Pflieger and R. Mettin

1. Spatial spectral analysis

Here we show some of the color long-term exposure images of SL and SCL, split in their red-green-blue (RGB) channels, to make a rudimentary spectral analysis of the spatial features. We suppose that SL sodium (Na*) emission at 589 nm is mainly contained in the red (R) channel and to some part in the green (G) channel, while SL continuum rises to the blue and will strongly show up in the green and blue (B) channel. Luminol (~380 … 600 nm) should activate essentially the blue channel and partly the green channel, although a small spectral overlap of the long-wavelength tail of luminol emission and the red pixel sensitivity exists; see the spectral diagram in Fig. S.0. Since we have no data on electrical crosstalk between channels, we assume that this might be neglected. The main results are:

- Na* emission zones can coincide or overlap with blue (continuum) emission.

- Na* emission zones can also be free of significant blue (continuum) emission.

- Neighboring, but separate individual bubbles within one bubble cloud can emit Na* with or without continuum.

- Luminol emission reaches to the periphery of overall emission, where essentially no native SL appears. This is pointing to chemically active non-sonoluminescing bubbles in the periphery, or/and to a mechanism of convective transport (of OH radicals, excited luminol, or intermediates). Convection over scales of cm with flow speeds in the range of m/s would hint to lifetimes of intermediates in the range of 10 ms or more.

Luminol emission spectrum from:
https://chem.libretexts.org/Bookshelves/Physical_and_Theoretical_Chemistry_Textbook_Maps/Supplemental_Modules_(Physical_and_Theoretical_Chemistry)/Spectroscopy/Electronic_Spectroscopy/Radiative_Decay/Chemiluminescence

Camera RGB spectral sensitivity from:
https://maxmax.com/faq/camera-tech/spectral-response/nikon-d700-study

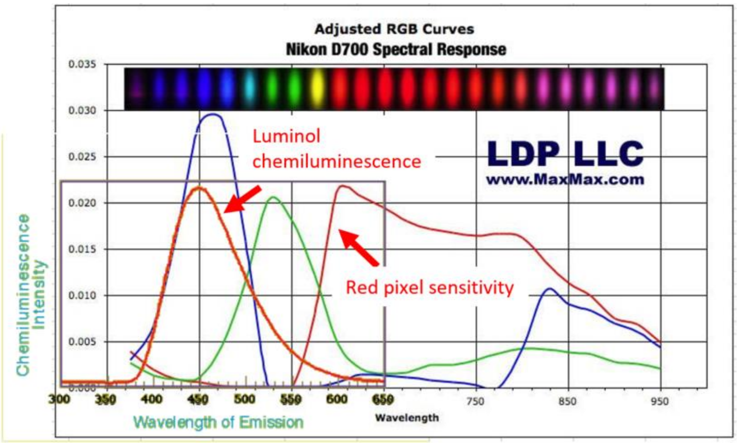

Figure S.0: Comparison of the standard luminol emission spectrum (according to chem.libretexts.org) and the spectral sensitivity of the camera Nikon D700 (by www.maxmax.com).

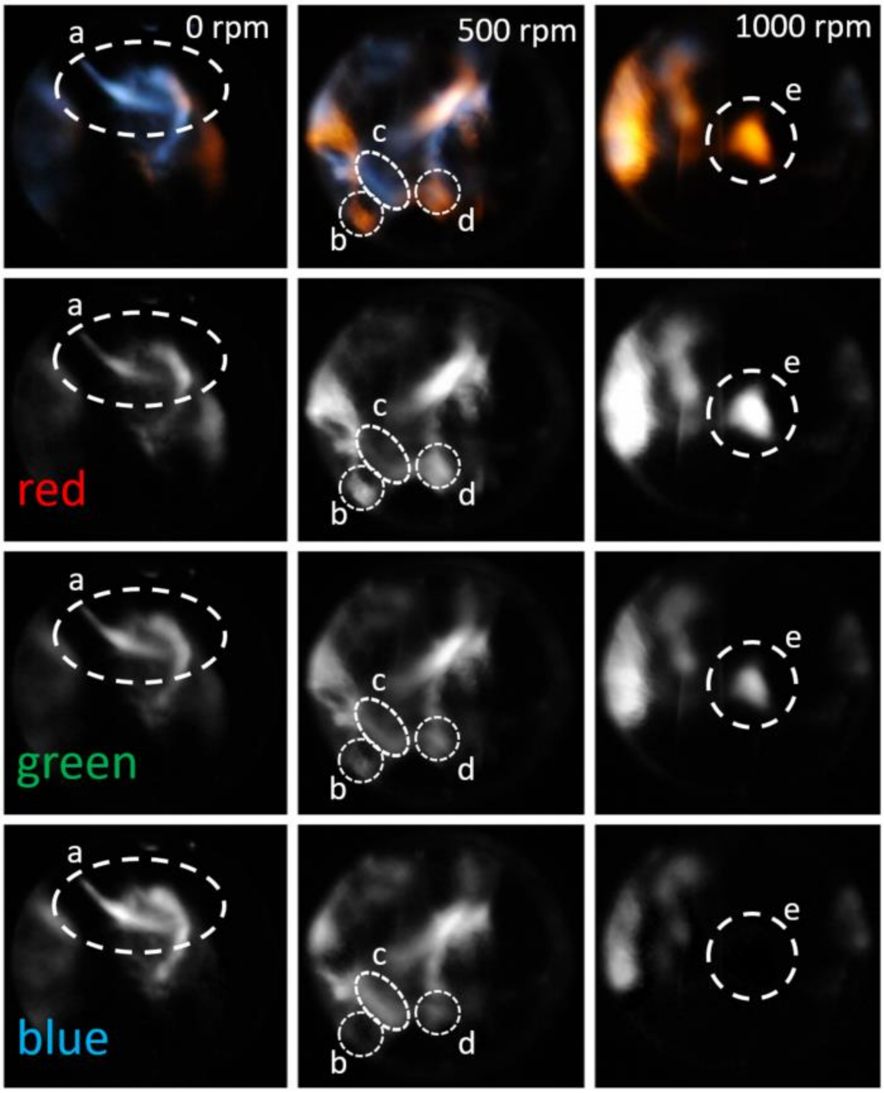

Figure S.1: SL in aqueous 3 M NaCl solution and Kr sparging, setup B, 36.5 kHz at 15 W, stirring rates indicated (from Fig. 2b). The red (R), green (G) and blue (B) channels of the color image are plotted as greyscale intensity images. R, G and B represent the long wavelength, central wavelength, and short wavelength parts of the visible spectrum. Regions of interest are encircled. The blue region in the left column (a) shows a strong blue component, a medium strong green component, and a weak red component. This is consistent with a continuous spectrum that rises to the blue without strong Na* signal. The red-blue region encircled in the center column shows mixed profiles: the left red feature (b) has no blue component, the right red feature (d) has a weak blue component, and the central blue feature (c) has also a red component. The circle in the right column shows a region (e) that has dominant Na* emission and no perceivable blue component. The stirring obviously shifts the emission towards strong and dominant Na* components.

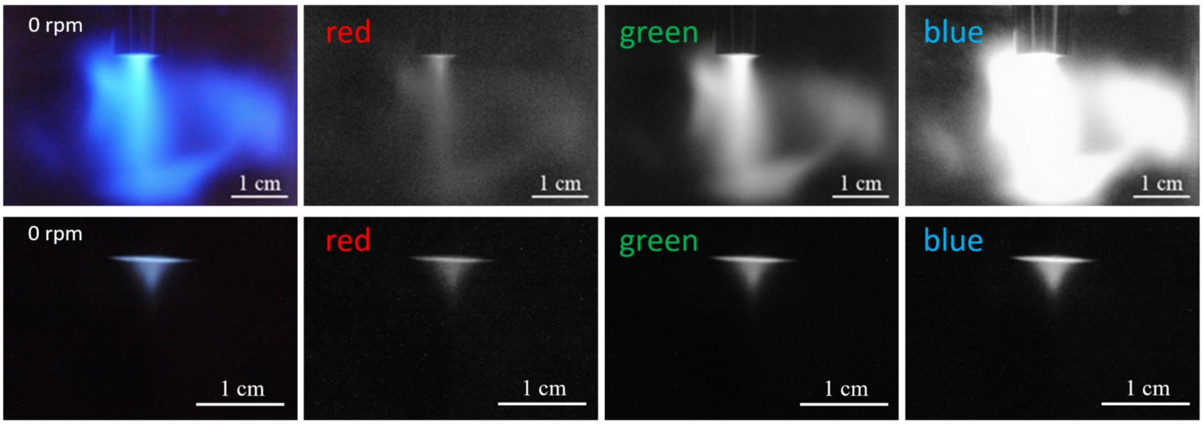

Figure S.2: Top row: SCL + SL from luminol aqueous solution and Ar, setup A, no stirring (from Fig. 6): very weak red component, medium strong green component, strongly dominant blue component. The red channel should represent native SL (mainly near the sonotrode tip), but apparently also contains some part of the long-wavelength tail of the luminol emission, and possibly pixel crosstalk and noise. Bottom row: Comparison of native SL in DI water and Ar, exposure 5 min. The emission intensity rises from red to the blue, and it is confined to the sonotrode tip and a small cone below. This confirms luminol emission far from native SL emissions.

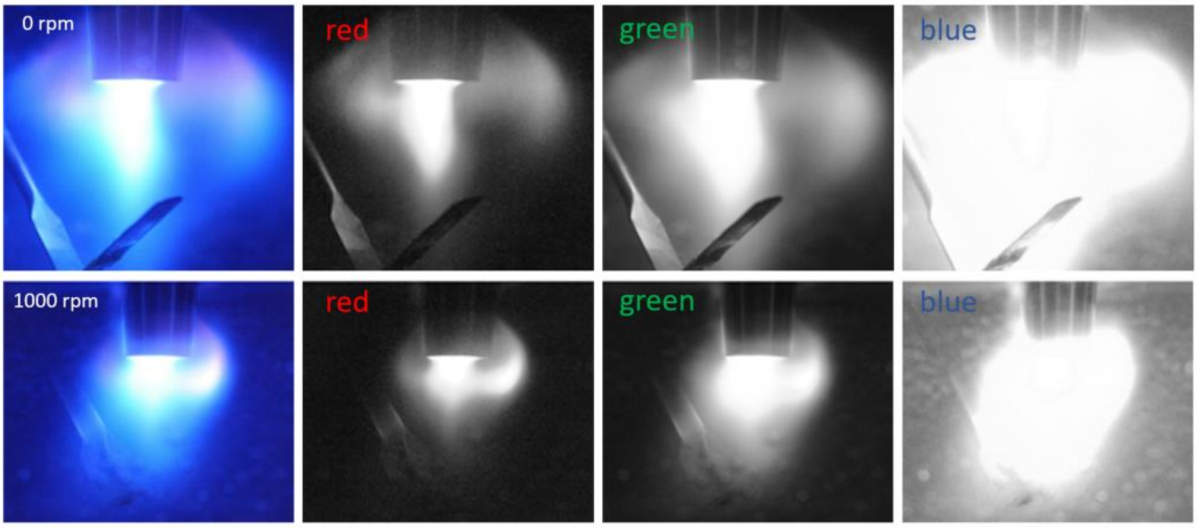

Figure S.3: SL + SCL in luminol aqueous solution with 3 M NaCl + Kr, setup A, stirring 0 rpm and 1000 rpm (from Fig. 6): Stirring confines the emission region and bends the jet to the right. Red emission is strong in the jet, weaker in wings at the sonotrode sides, and mainly absent in the lower periphery. Blue and green fills the full emission region, blue even to saturation. Blue without red should indicate OH production in chemically active bubbles or OH transport or excited luminol transport, since native SL should leave a detectable trace in the red channel, compare the bottom row of Fig. S.2 above.

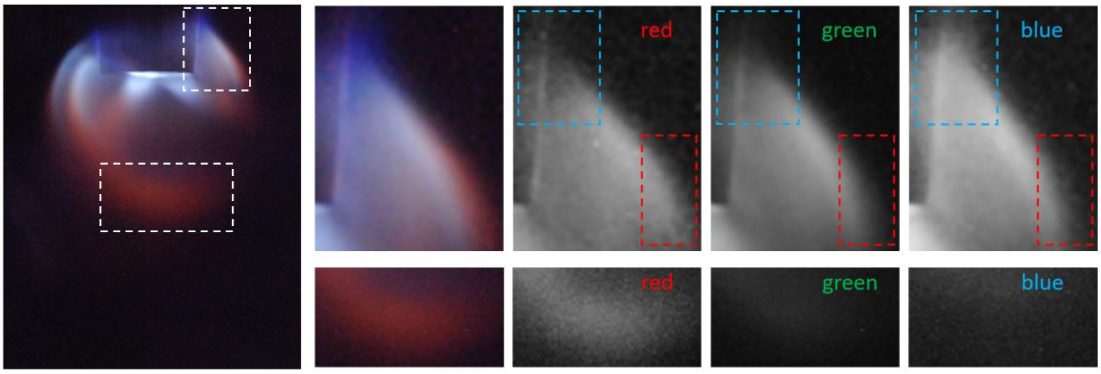

Figure S.4: SL + SCL in EG + luminol + 0.3 M NaCl + Ar, setup A, stirring at 1000 rpm (from Fig. 9a): Red emission occurs at the bottom border of the luminescent zone (left, lower white rectangle). RGB splitting reveals a dominant Na* emission with very weak blue and nearly no green components. Near the edge of the sonotrode tip (left, upper white rectangle), blue color with weak green and nearly no red occurs, which appears to origin from "pure" luminol emission. This zone is marked with a blue rectangle. Farther out (red rectangle), the blue disappears at the emission border and red dominates.

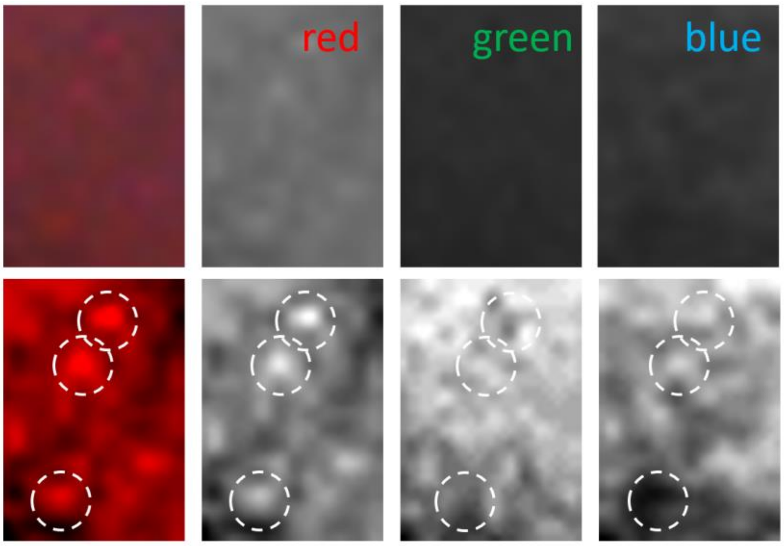

Figure S.5: Magnified detail of the red zone shown in Fig. S.4, bottom (EG + luminol + 0.3 M NaCl + Ar at 1000 rpm). The upper row shows the raw data and the RGB splitting, the lower row the respective images after contrast and brightness enhancement. Individual spots are supposed to reflect emissions from individual bubbles [S-1]. Some red spots in the upper part have counterparts in blue, i.e. the Na* line rides on a significant continuum. Other red spots like the one marked in the bottom have no correspondence to blue or green emission sites. This suggests a very low continuum emission for these bubbles, similar to the spectra of red emitting regions in acids or aqueous systems reported before for instance in [S-2, S-3, S-1]. Thus it appears like the bubble emissions within a cloud can have different individual spectral characteristics.

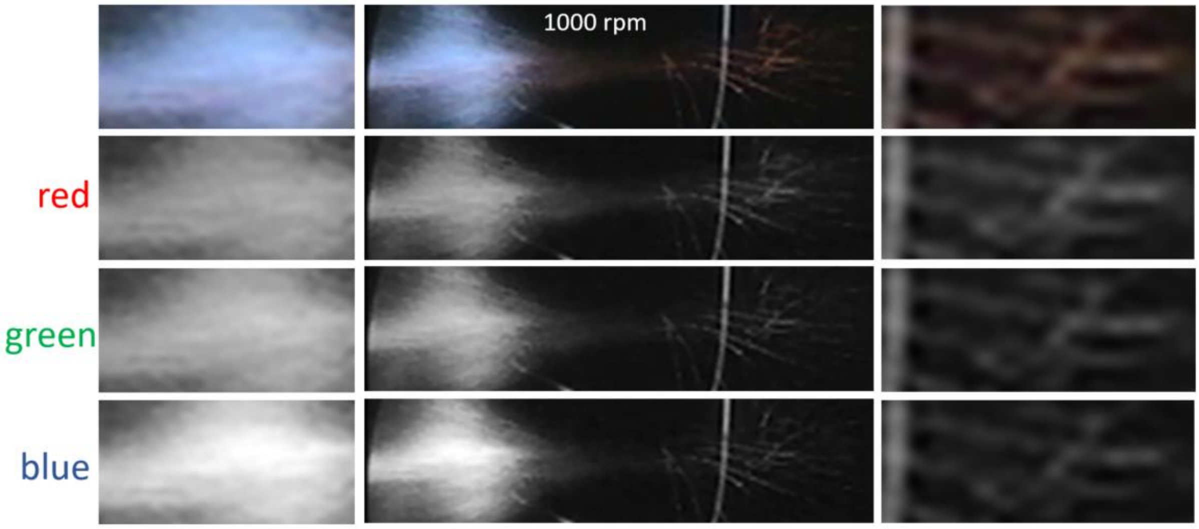

Figure S.6: Magnified details of the cloud region and the streak region Fig. 10c (setup B, PA + 0.5 M sodium phosphate + Kr at 1000 rpm). The upper row shows the raw data, and the lower rows the corresponding RGB channels. Left and right columns show magnifications of the center images. Both cloud and streaks emit all colors, the cloud stronger in the blue and the streaks stronger in the red.

2. Comparison of SL and SCL emission in the continuum band

To get a rough quantitative idea on the relation of native SL and luminol SCL at 0.1 mM concentration, we add data here for an experiment at 359 kHz, Fig. S7. The result shows that (for the used “continuum” band 450 nm – 480 nm) we see a 3-fold emission from luminol as compared to pure water. It remains to be shown that this result translates to the 20 kHz range, and that modifications of native SL by luminol molecules entering the bubble can be neglected. However, this gives some estimate of the relative contributions of SL and SCL.

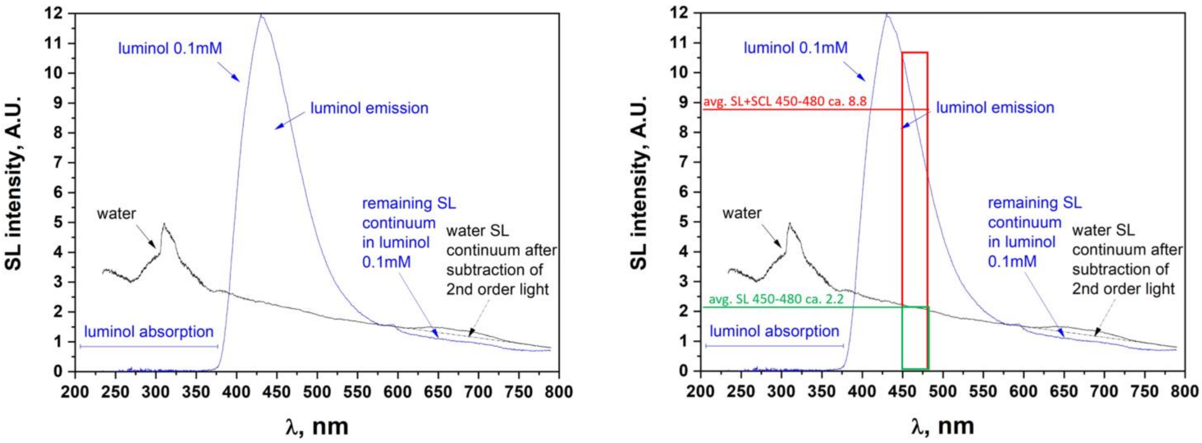

Figure S.7: Left: Pure water (black) and 0.1 mM aqueous luminol (blue) emissions under identical spectral observation conditions and sonication at 359 kHz. Right: The used “continuum” window of 450-480 nm is marked by rectangles. The approximate average intensities result in a 4-fold higher value for ”SCL + SL” than for ”SL” alone. This translates into 3-fold higher “continuum” intensity of SCL than SL.

3. Spectral components and dissolved air remnants

The appearance of nitrogen and carbon components in the SL spectra of liquids that do not contain N or C can occur by air in the bubbles. This holds true even in cases where the liquid is (partly) degassed from air and sparged by another gas (e.g. noble gas). The reason is then an insufficient degassing of air previous to sonication and sparging. For liquids with higher viscosity (like EG or PA), the full degassing is difficult and also difficult to check. Thus we attribute the occurrence of such features in our experiments with EG and PA to air remnants, even after very long vacuum degassing under stirring before the run, and with partial noble gas sparging during the run. An example for emergence and later disappearance of NH and CN in water is shown in Fig. S.8. The disappearance of NH and CN features seems to indicate the final decline of dissolved air, degassed by the action of sonication and cavitation.

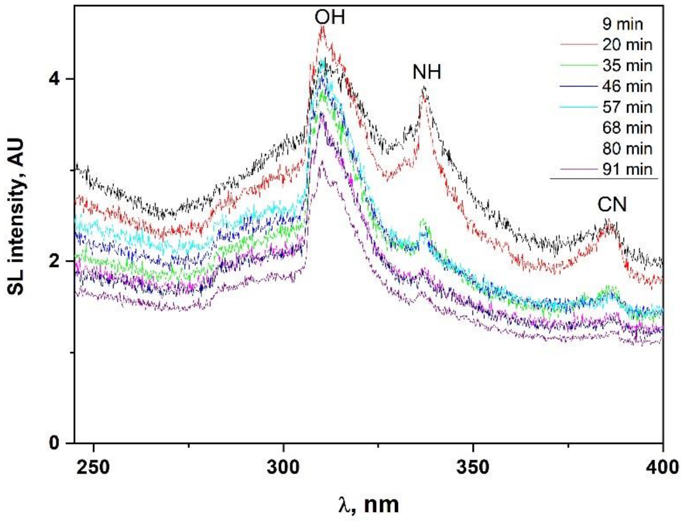

Figure S.8: SL spectra of water sonicated at 200 kHz, initially saturated with air, and submitted to an Ar flow started at the same time as sonication. Initially (i.e. in the absence of Ar) the spectrum consists of a continuum only. After a few minutes of concomitant sonication and Ar sparging, peaks start to appear, that are attributed to OH, NH and CN. NH and CN indicate the presence of air in the solution, since they were formed from N_2 and CO_2.

References

[S-1] Thiemann, A., Holsteyns, F., Cairós, C. and Mettin, R., 2017. Sonoluminescence and dynamics of cavitation bubble populations in sulfuric acid. Ultrasonics sonochemistry, 34, pp.663-676. https://doi.org/10.1016/j.ultsonch.2016.06.013

[S-2] Hatanaka, S.I., Hayashi, S. and Choi, P.K., 2010. Sonoluminescence of alkali-metal atoms in sulfuric acid: Comparison with that in water. Japanese Journal of Applied Physics, 49(7S), p.07HE01. https://doi.org/10.1143/JJAP.49.07HE01

[S-3] Cairós, C., Schneider, J., Pflieger, R. and Mettin, R., 2014. Effects of argon sparging rate, ultrasonic power, and frequency on multibubble sonoluminescence spectra and bubble dynamics in NaCl aqueous solutions. Ultrasonics sonochemistry, 21(6), pp.2044-2051. https://doi.org/10.1016/j.ultsonch.2014.03.006
